# Sialidase NEU3 Dynamically Associates to Different Membrane Domains Specifically Modifying Their Ganglioside Pattern and Triggering Akt Phosphorylation

**DOI:** 10.1371/journal.pone.0099405

**Published:** 2014-06-12

**Authors:** Dario Bonardi, Nadia Papini, Mario Pasini, Loredana Dileo, Flavia Orizio, Eugenio Monti, Luigi Caimi, Bruno Venerando, Roberto Bresciani

**Affiliations:** 1 Department of Molecular and Translational Medicine, University of Brescia, Brescia, Italy; 2 Department of Medical Biotechnology and Translational Medicine, University of Milan, Milan, Italy; University of Insubria, Italy

## Abstract

Lipid rafts are known to regulate several membrane functions such as signaling, trafficking and cellular adhesion. The local enrichment in sphingolipids and cholesterol together with the low protein content allows their separation by density gradient flotation after extraction with non-ionic detergent at low temperature. These structures are also referred to as detergent resistant membranes (DRM). Among sphingolipids, gangliosides play important roles in different biological events, including signal transduction and tumorigenesis. Sialidase NEU3 shows high enzymatic specificity toward gangliosides. Moreover, the enzyme is present both at the cell surface and in endosomal structures and cofractionates with caveolin. Although changes in the expression level of NEU3 have been correlated to different tumors, little is known about the precise distribution of the protein and its ability in modifying the ganglioside composition of DRM and non-DRM, thus regulating intracellular events. By means of inducible expression cell system we found that i) newly synthesized NEU3 is initially associated to non-DRM; ii) at steady state the protein is equally distributed between the two membrane subcompartments, i.e., DRM and non-DRM; iii) NEU3 is degraded via the proteasomal pathway; iv) the enzyme specifically modifies the ganglioside composition of the membrane areas where it resides; and v) NEU3 triggers phosphorylation of Akt, even in absence of exogenously administered EGF. Taken together our data demonstrate that NEU3 regulates the DRM ganglioside content and it can be considered as a modulator of Akt phosphorylation, further supporting the role of this enzyme in cancer and tumorigenesis.

## Introduction

The lipid and protein components of cellular membranes can be laterally segregated, giving rise to specialized functional structures named lipid rafts, known to play important roles in regulating several membrane functions such as signaling, trafficking and cellular adhesion [1]. Lipid rafts represent dynamic liquid-liquid immiscibility entities, thus membrane subcompartmentalization. They are dynamic fluctuating nanoscale assemblies of sphingolipids, cholesterol and proteins that dissociate and associate usually on a rapid timescale. The relatively few proteins present in these areas are mainly represented by GPI-anchored proteins, members of the Src-family tyrosine kinases, cholesterol-binding proteins such as caveolins, and heterotrimeric G-proteins [1], [2]. The high concentration in sphingolipids and cholesterol together with the low protein content in lipid rafts determine their low density and insolubility in presence of non-ionic detergents, thus they are also referred as detergent resistant membranes (DRM) [3]. Detergent treatment at low temperature allows the solubilization of the “liquid disordered” bilayer, generally without interfering with the structure and composition of the “liquid-ordered” sub-compartment.

Gangliosides are sialic acid containing sphingolipids present in the outer leaflet of the membrane, known to play important roles in different biological events, including cell-to-cell, cell-to-environment interactions and signal transduction. Among gangliosides, GM3 binds to EGFR, regulating its activity [4], [5]. The membrane ganglioside pattern is the result of the correct balance between their synthesis, mediated by sialyl-transferases, and degradation, which is mediated by sialidases (NEUs). Among sialidases, NEU3 was initially identified as the membrane-associated member of the sialidase family enzyme, showing high enzymatic specificity toward gangliosides, especially GM3 and GD1a [6]–[9]. Moreover, NEU3 is a peripheral membrane protein present both at the cell surface and in endosomal structures [10], known to cofractionate with caveolin [11]. Although up-regulation of NEU3 has been correlated to different tumors [12], [13], little is known about the precise distribution of the protein in relation to membrane sub-compartments, i.e. DRM and non-DRM, and its ability in modifying the ganglioside composition of these membrane compartments, thus regulating intracellular events.

By means of inducible expression cell system we found that i) newly synthesized NEU3 is initially associated to non-DRM; ii) at steady state the protein is equally distributed between the two membrane subcompartments; iii) NEU3 is degraded via the proteasomal pathway; iv) the enzyme specifically modifies the ganglioside composition of the membrane areas where it resides, and v) NEU3 triggers phosphorylation of Akt, even in absence of exogenously administered EGF.

Taken together our data indicate that sialidase NEU3 is the enzyme responsible for the modifications of the ganglioside composition both in DRM and in non-DRM and can be considered as a modulator of Akt phosphorylation, further supporting its role in cancer and tumorigenesis.

## Materials and Methods

### Chemicals and reagents

All chemicals are molecular biology-grade (SIGMA-Aldrich) unless specified, common solvents were from Merck. High performance silica gel-precoated thin-layer plates (HPTLC Kieselgel 60) were purchased from Merck GmbH. [3-^3^H]Sphingosine (specific radioactivity 19.8 Ci/mmol) was provided by PerkinElmer Life Sciences.

### Antibodies

For immunoblotting experiments, the following primary antibodies were used: rabbit anti-HA 1∶1000 (Sigma), mouse anti-TfR (transferrin receptor) 1∶400 (Invitrogen), rabbit anti-Cav-1 (caveolin-1) 1∶250 (Santa Cruz Biotechnology), mouse anti-α-tubulin 1∶16000 (Sigma), mouse anti-ERK1/2 1∶1000 (Santa Cruz Biotechnology) and mouse anti-phosphoERK1/2 (Tyr204) 1∶1000 (Santa Cruz Biotechnology), rabbit anti-Akt1/2/3 1∶4000 (Cell Signaling) and rabbit anti-phosphoAkt (Thr308) 1∶4000 (Cell Signaling). HRP (horseradish peroxidase)-conjugated donkey anti-rabbit and sheep anti-mouse secondary antibodies 1∶5000 (GE Healthcare) were used.

### Plasmid preparation

pcDNA1/Amp MmNEU3-HA, gently provided by Dr. T. Miyagi (Institute of Molecular Biomembrane and Glycobiology, Tohoku Pharmaceutical University, Sendai, Miyagi, Japan) and already used in previous works, was mutagenized in order to insert downstream the HA-epitope the following restriction sites: *BglII*, *SacII* and *XbaI*. pEGFP-C2 (Clontech) was used to amplify the GFP coding sequence by PCR using amplification oligonucleotides carrying a *BglII* site at 5′ and a *SacII* site at 3′. The amplified GFP sequence was initially subcloned into the mutagenized pcDNA1/MmNEU3-HA and subsequently the whole ORF MmNEU3–HA-GFP was excised and subcloned into the pUHD 10.3 plasmid [14] using the 5′ *EcoRI* site and the 3′ *XbaI* site. pUHD 10.3/MmNEU3-HA-GFP was then mutagenized in order to prepare a second NEU3 expressing plasmid without GFP. For this purpose we inserted a *BglII* restriction site downstream the GFP epitope and used it for the excision of GFP and subsequent ligation, obtaining the pUHD 10.3/MmNEU3-HA plasmid.

### Cell culture and transfection

Hela tTA2 cells, over-expressing the Tetracycline transactivator (Tet-OFF), were gently provided by S. Schmidt (Scripps Institute, S. Diego, CA, USA) [15] and were cultured in high glucose DMEM (Dulbecco's modified Eagle's medium) (EuroClone) containing 4 mM L-glutamine, 100 units/ml penicillin, 100 µg/ml streptomycin and 10% (v/v) fetal bovine serum (FBS) and 0.25 mg/ml G418, and were maintained at 37°C and 5% CO_2_ in a humidified incubator. Cells were stably co-transfected with pUHD 10.3 NEU3–HA-GFP and pSV2-pac or pUHD 10.3 NEU3-HA and pHygro plasmids in a ratio 40∶1, respectively. Mock cells were obtained by transfection with pSV2-pac or pHygro plasmids alone. Transfection was performed in serum-free medium OptiMEM (Life Technologies) employing FuGENE 6 (Roche). After 6 h transfection the medium was changed to complete medium (DMEM+10% FBS+0.25 mg/ml G418) and after further 48 h resistant clones were selected by addition of puromycin (2 µg/ml) or hygromycin (200 µg/ml) to the growth medium. Puromycin- and hygromycin-resistant clones were isolated and tested for NEU3 enzymatic activity and for intensity of GFP fluorescence, the latter only for NEU-HA-GFP clones; the most homogeneous and enzymatically active clones were used in this study and were cultured in high glucose DMEM containing 4 mM L-glutamine, 100 units/ml penicillin, 100 µg/ml streptomycin, 10% (v/v) fetal bovine serum (FBS), 0.25 mg/ml G418, 0.5 µg/ml puromycin or 200 µg/ml hygromycin, and 1 ng/ml doxycycline (dox). Medium containing dox was replaced every 48 hours. Expression of NEU3-HA-GFP and NEU3-HA was achieved by removing dox from culture medium.

### Sialidase activity assay

The enzymatic activity of HeLa tTA2 NEU3-HA-GFP and HeLa tTA2 NEU3-HA cells, treated or not with dox, was determined as described previously [7] using 0.1 mM 4 MU-NeuAc (4-methylumbelliferyl-*N*-acetyl-α-D-neuraminic acid) as substrate.

### Immunoblotting

Proteins were separated by SDS/10% PAGE and transferred to a Hybond-P PVDF membrane (GE Healthcare). Membranes were then blocked with 5% (w/v) non-fat dried skimmed milk in PBS, washed three times with PBS containing 0.1% Tween 20 (PBST) and incubated with primary antibody diluted in PBST containing 1% (w/v) non-fat dried skimmed milk for 1 h at room temperature. After three washes with PBST, membranes were incubated with HRP-conjugated secondary antibody diluted in PBST for 45 min at room temperature. Detection of the immunocomplexes was performed using the enhanced chemiluminescence-based system (SuperSignal West Pico Chemiluminescent Substrate; Pierce), followed by densitometric analysis using GelPro 3.1 software (Media Cybernetics).

### RNA extraction and real-time RT-PCR

Total RNA was extracted from cells using the RNeasy mini kit (Qiagen), according to the manufacturer's protocol. The iScript cDNA Synthesis Kit (Bio-Rad Laboratories) was used to reverse-transcribe 0.8 µg of RNA. Real time PCR was performed by the iCycler thermal cycler (Bio-Rad Laboratories) using cDNA corresponding to 10 ng of total RNA as template. PCR mixture included 0.2 µM primers, 50 mM KCl, 20 mM Tris/HCl pH 8.4, 0.8 mM dNTPs, 0.7 U iTaq DNA Polymerase, 3 mM MgCl_2_, and SYBR Green (iQ SYBR Green Supermix from Bio-Rad Laboratories) in a final volume of 20 µl. Amplification and real time data acquisition were performed using the following cycle conditions: initial denaturation at 95°C for 3 min, followed by 40 cycles of 10 s at 95°C and 30 s at 58°C. The fold change in expression of the different genes in NEU3-HA-GFP overexpressing cells compared with Mock cells was normalized to the expression of glyceraldeide 3-phosphate dehydrogenase (GAPDH) mRNA and was calculated by the equation 2^−ΔΔCt^ with iQ5 Software Version 2.0 (Bio-Rad). All reactions were performed in triplicate. The primers used were: *HsNEU1*: Fw 5′-CCTGGATATTGGCACTGAA-3′ and Rev 5′-CATCGCTGAGGAGACAGAAG-3′; *HsNEU3*: Fw 5′-TGAGGATTGGGCAGTTGG-3′ and Rev 5′-CCCGCACACAGATGAAGAA-3′; *HsGAPDH*: Fw 5′-AGGGCTGCTTTTAACTCTGG-3′ and Rev 5′-CATGGGTGGAATCATATTGG-3′. The accuracy was monitored by the analysis of the melting curves.

### Metabolic labeling of cell sphingolipids

[3-^3^H]Sphingosine dissolved in ethanol was transferred into a glass sterile tube and dried under a nitrogen stream; the residue was then dissolved in an appropriate volume of pre-warmed DMEM+10% FBS to obtain a final concentration of 0.25 µCi/100 mm dish (corresponding to 2.6×10^−9^ M). OFF and ON HeLa tTA2 NEU3-HA-GFP cells were incubated with DMEM+10% FBS containing [3-^3^H]Sphingosine. After a 2 h incubation, the medium was removed and cells were chased for 48 h with DMEM+10% FBS, always containing or not dox, in order to reach the metabolic steady state. This condition was previously established with ad hoc experiments. At the end of chase, cells were washed and harvested in ice-cold phosphate-buffered saline (PBS) by scraping. Cell suspensions were frozen and then lyophilized.

### Extraction and analysis of radioactive lipids

Total lipids from lyophilized cells were extracted twice with chloroform/methanol/water 20∶10∶1 (v/v). Lipid extracts were dried under a nitrogen steam, dissolved in chloroform/methanol 2∶1 (v/v) and subjected to a two-phase partitioning in chloroform/methanol 2∶1 and 20% (v/v) water. The aqueous and organic phases obtained were counted for radioactivity and analyzed by HPTLC. [^3^H]Sphingolipids of organic phase were separated using the solvent system chloroform/methanol/water 55∶20∶3 (v/v). The solvent system chloroform/methanol/0.2% aqueous CaCl_2_ 60∶40∶9 (v/v) was employed to analyze [^3^Η]Sphingolipids of aqueous phase. Radioactive lipids were visualized with a Beta-Imager 2000 (Biospace, Paris, France) and identified by comparison with radiolabeled standards. The radioactivity associated with individual lipids was determined with the specific β-Vision software (Biospace, Paris, France).

### Preparation of soluble and membrane-associated protein fractions

DRM preparation was adapted from a previously described procedure with minor modifications [16]. Adherent cells were washed twice with cold PBS, scraped from the dishes, gently resuspended in TNE buffer (20 mM Tris-HCl pH 7.5, 150 mM NaCl, 1 mM EDTA) supplemented with cold 1% Triton X-100 (TNE-TX) and a protease inhibitor cocktail, and extracted for 30 min on ice. Cell extracts were further processed in two different manners, as described below.

#### Crude separation

One third of the cell extracts was withdrawn and kept as total fraction, and the residual two thirds was centrifuged at 100,000×*g* for 1 h with a Beckman 50Ti rotor. Pelleted material was resuspended in the same volume of the supernatant with TNE-TX buffer. Aliquots of the total, pellet and supernatant fractions were used for Western blot analysis [17].

#### OptiPrep Gradient Separation

Cell extracts were adjusted to 40% v/v OptiPrep in a final volume of 1.1 ml, placed at the bottom of a 4-ml ultracentrifuge tube and overlaid with 2 ml 30% OptiPrep and 0.9 ml 5% Optiprep step gradient. All dilutions were performed in TNE-TX buffer. After ultracentrifugation (170,000×g, 4°C, 4 h; Beckman SW50.1 rotor), fractions of 500 µl were collected from the top and processed for further analyses [3], [18].

### Sphingolipid pattern modification during time-dependent sialidase expression

To assess NEU3-HA-GFP and NEU3-HA activity toward gangliosides of DRM and non-DRM areas during time-dependent sialidase expression, we performed a metabolic labelling with [3-^3^H]Sphingosine and then DRM and non-DRM fractions were separated on a Optiprep density gradient. In brief, NEU3-HA-GFP and NEU3-HA cells in presence of dox were treated with [3-^3^H]Sphingosine according to the usual protocol. For each sample, 5 dishes each of 100 mm diameter were used. After a 24 h chase, dox was removed and cells were cultured for different time periods. At the end of incubation cells were harvested and lysed in TNE-TX containing a protease inhibitor cocktail, for 20 min on ice. Cell lysates were adjusted to 40% v/v OptiPrep in a final volume of 3.3 ml, placed at the bottom of ultracentrifuge tube and overlaid with 6 ml 30% OptiPrep and 2.7 ml 5% Optiprep step gradient. After ultracentrifugation (170,000×g, 4°C, 4 h; Beckman SW41 Ti rotor), fractions of 1.5 ml, except for the first fraction (1.2 ml), were collected from the top to the bottom of the gradient, 4/5 volume of each fraction was employed to analyze [^3^H]Sphingolipid pattern. The rest was processed for sialidase activity and western blotting analyses. Ganglioside and other sphingolipids were extracted, separated and quantified as described previously.

### Cell stimulation with EGF

OFF NEU3-HA-GFP cells were plated and maintained in serum-free medium for 24 h before harvesting. At the same time, expression of NEU3-HA-GFP was allowed for different time periods (8, 24, 48 and 72 h). Before harvesting cells were stimulated or not with 100 ng/ml EGF for 10 min. After stimulation cells were washed twice with cold PBS and then lysed for 15 min at 4°C in lysis buffer (25 mM Tris-HCl pH 7.4, 150 mM NaCl, 5 mM EDTA, 20 mM NaF, 1 mM Na_3_VO_4_, 0.5% (v/v) NP40, 10 µg/ml leupeptin, 10 µg/ml aprotinin, 1 µg/ml pepstatin A). Insoluble material was removed by centrifugation at 13,000×g for 10 , supernatants were collected and assayed for protein concentration with Coomassie Protein Assay (Pierce). Western blot analysis for the different intracellular signaling molecules was performed using aliquots deriving from the same cell extracts.

### Confocal microscopy analysis

Subcellular distribution analysis of NEU3-HA-GFP was performed as described previously [7], with minor modifications. ON HeLa tTA2 NEU3-HA-GFP expressing cells were seeded onto glass coverslips and after 48 h were grown for 8 h in medium containing dox supplemented or not with 5 µM MG132, or for 6 h in medium containing dox and 5 µM MG132, followed by 2 h chase in medium without MG132. Cells were then washed three times with PBS containing 1 mM MgCl_2_ and 1 mM CaCl_2_ (PBS^++^) and fixed with 3% (w/v) paraformaldehyde containing 2% sucrose in PBS^++^ for 15 min at room temperature. Paraformaldehyde was then quenched by incubating samples with 50 mM NH_4_Cl in PBS^++^ for 15 min. Finally, specimens were washed three times with PBS^++^, mounted using DakoCytomation Fluorescent Mounting Medium and analyzed with confocal system LSM-510 META (Carl Zeiss). Images were processed with LSM Image Browser (Carl Zeiss) and Adobe Photoshop software.

### Statistical data analysis

Data are the means ± standard deviations (S.D.). Statistical analysis were made using unpaired Student's *t* test. Significance was attribute at the 95% level of confidence (*P*-value <0.05).

## Results

### Inducible expression of NEU3-HA-GFP

**Figure 1:**
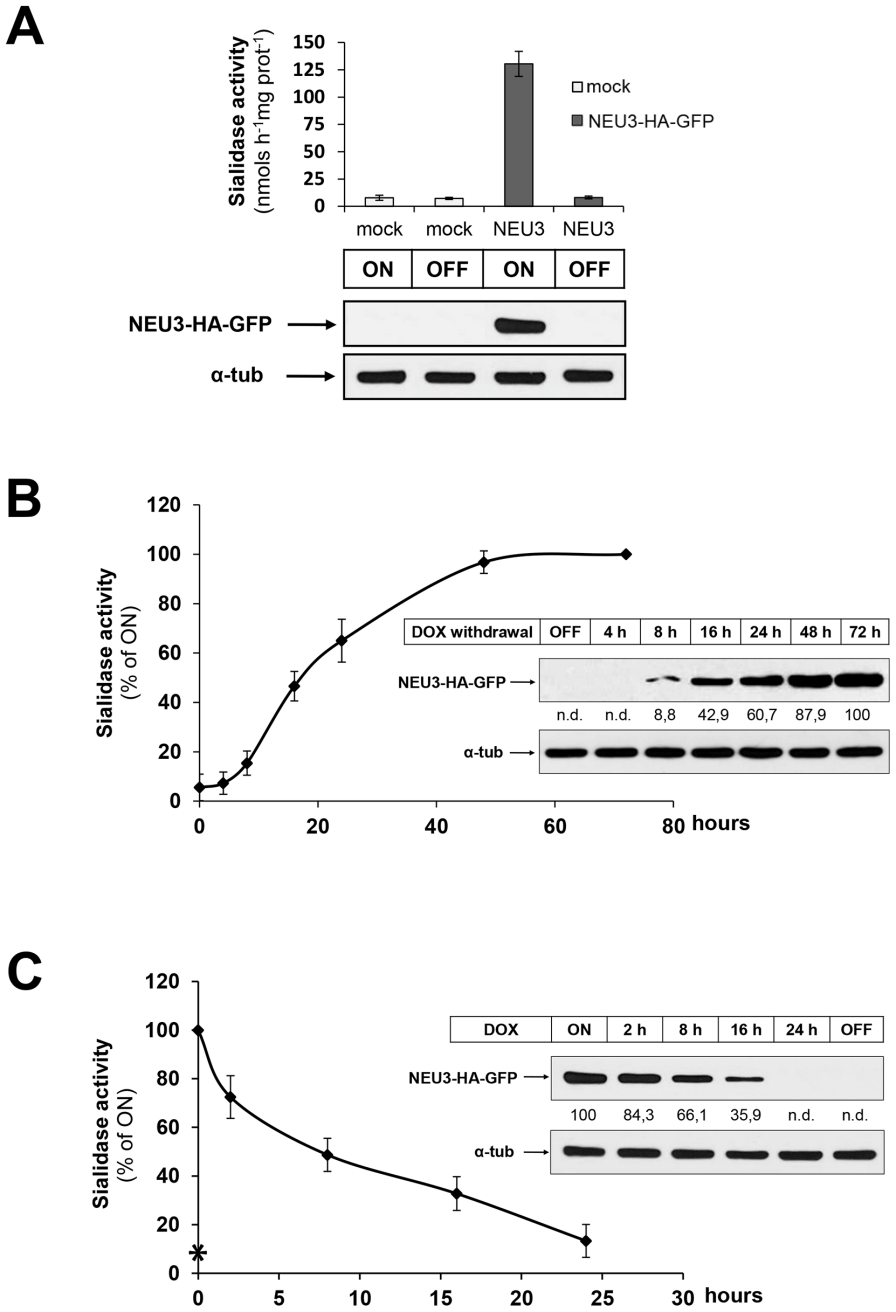
Characterization of the inducible expression cell model HeLa tTA2 NEU3-HA-GFP. (A) HeLa tTA2 cells stably transfected with pUHD 10.3 NEU3-HA-GFP or with the empty vector (mock) were grown for 7 days in absence (ON) or presence (OFF) of dox. Cell homogenates were analyzed for sialidase activity (values are given as mean +/− SD and represent the mean of 5 independent experiments) and for NEU3-HA-GFP expression using anti-HA primary antibody. Alpha-tubulin was detected with specific primary antibody in order to normalize NEU3-HA-GFP signal. (B) OFF HeLa tTA2 NEU3-HA-GFP were plated and grown in absence of dox for the indicated time periods. Cell homogenates were analyzed for sialidase activity (values refer to ON cells and represent the mean +/− SD of 4 independent experiments) and NEU3-HA-GFP expression using anti-HA primary antibody. NEU3-HA-GFP optical density was normalized to alpha-tubulin as indicated in (A) and values are given. (C) ON HeLa tTA2 NEU3-HA-GFP were plated and grown in presence of dox for the indicated time periods. Cell homogenates were analyzed for sialidase activity and NEU3-HA-GFP expression as indicated in (B). Asterisk indicates the sialidase activity measured in OFF cells.

In order to study the membrane association and distribution of sialidase NEU3 during its biosynthesis we expressed the murine enzyme protein under controlled experimental conditions, taking advantage of the tetracycline inducible expressing system. We introduced the EGFP coding sequence at the C-terminus of the already available plasmid pcDNA1-NEU3-HA expressing the mouse sialidase NEU3 tagged to the HA epitope [7], [10], and the resulting ORF of the chimeric protein NEU3-HA-GFP was then subcloned in the pUHD-10.3 plasmid and used to stably transfect HeLa tTA2 cells, expressing the tetracycline-controlled transactivator protein tTA. This is expected to result in the cell model HeLa tTA2 NEU3-HA-GFP where the expression of the enzyme is constitutive in absence of the tetracycline-derivative doxycycline (dox) and, on the other hand, expression of the chimeric protein would be null in presence of dox. To verify these aspects, HeLa tTA2 NEU3-HA-GFP transfected cells cultivated in presence of 1 ng/ml dox were plated and grown for further 7 days in absence (ON) or presence (OFF) of dox and tested for their sialidase activity and protein expression. Crude homogenates deriving from ON cells showed a 16-fold increase in sialidase activity measured with the artificial substrate 4-MU-NeuAc and compared to the endogenous enzymatic activity measured in mock-transfected HeLa tTA2 cells, corresponding to 130.39+/−11.49 and 7.88+/−2.32 nmols h^−1^ mg Prot^−1^, respectively (Figure 1A). As expected, crude homogenates deriving from the same clone and grown in presence of dox (OFF) showed sialidase activity comparable to mock HeLa tTA2 cells, indicating that expression of NEU3-HA-GFP is indeed regulated by the presence of dox in the growth medium. In order to confirm that the increase in sialidase activity results from the overexpression of the chimeric protein, equal protein amounts of cell extracts were analyzed by western blot using anti-HA primary antibody. As shown in Figure 1A, a protein band of the expected molecular weight of the NEU3-HA-GFP chimera protein and corresponding to 78 kDa was detected only in cell extracts deriving from ON HeLa tTA2 NEU3-HA-GFP cells. These data demonstrate that in our cell system, expression of NEU3-HA-GFP is strictly under the control of the tetracycline promoter.

This finding prompted us to further characterize our cell system and to investigate the time course of NEU3-HA-GFP expression. OFF HeLa tTA2 NEU3-HA-GFP cells were plated and grown for different time periods under permissive conditions. Removal of dox resulted in the increase of sialidase activity in the cell homogenates (Figure 1B). Under these experimental conditions, after 18 h expression cells reached 50% of sialidase activity measured in ON cells while full activity was achieved after 72 h expression. Is worth to note that the increase in sialidase activity correlates with densitometric analysis of the specific protein bands detected in cell homogenates, normalized to the alpha-tubulin signal of each sample (Figure 1B, inset). We then investigated NEU3-HA-GFP degradation kinetic and for this purpose, ON HeLa tTA2 NEU3-HA-GFP cells were plated and cultivated for different time periods in presence of dox. A fast decrease in sialidase activity in total homogenates was observed (Figure 1C), correlating with the densitometric analysis of the immunoreactive bands detected by western-blot (Figure 1C, inset). More detailed, after 24 h in presence of dox, sialidase activity was almost comparable to the residual endogenous activity measured in OFF cells. This approach allowed us also to estimate the approximate half-life of the chimera protein that resulted to be about 8 h.

Taken together these data demonstrate that the chimera protein NEU3-HA-GFP is fully active and that in our cell system its expression is tightly regulated by the presence of dox in the growth medium.

### Expression of NEU3-HA-GFP specifically modifies the cellular ganglioside composition

**Figure 2:**
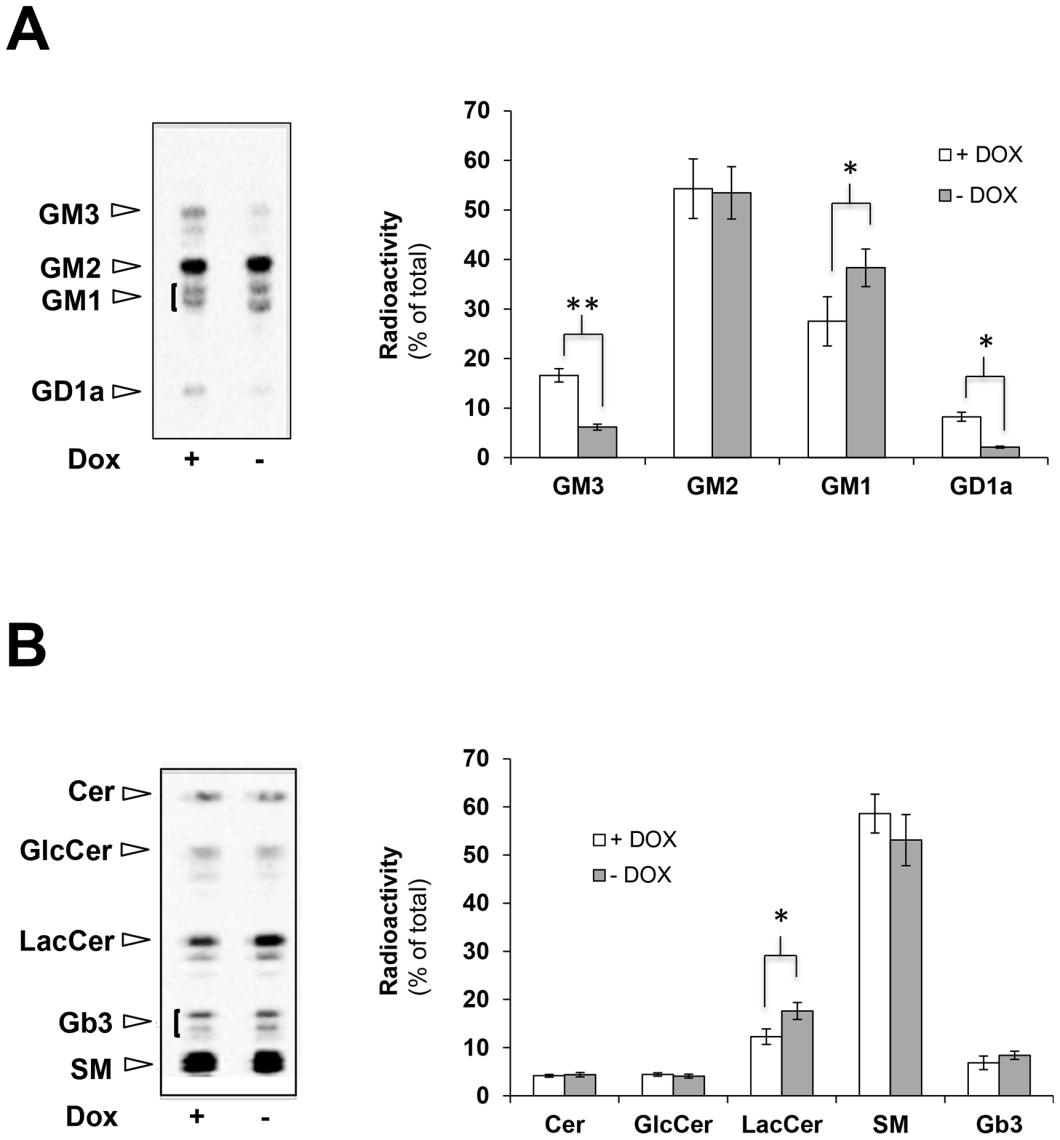
Expression of NEU3 results in the modification of the cellular ganglioside composition. OFF HeLa tTA2 NEU3-HA-GFP cells were plated and further grown for 72 h in absence (ON) or presence (OFF) of dox. Cells were then metabolically labeled for 2 h with [^3^H]-sphigosine and chased for 48 h. Cell lipids were extracted and gangliosides (A) and non-ganglioside sphingolipids (B) were separated by HPTLC and visualized (left, Beta-Imager 2000) and associated radioactivity was determined (right, Beta-Vison). Values are given as percentage of total radioactivity and represent the means ± S.D. of 5 independent experiments. *p<0.05; **p<0.01.

Since NEU3 is known as the sialidase family member that specifically exerts its enzymatic activity toward gangliosides [6], [19], we then analyzed the sphingolipid pattern of [^3^H]-Sphigosine metabolically labeled cells. OFF HeLa tTA2 NEU3-HA-GFP cells were plated and grown for 72 h in absence or presence of dox. Cells were then metabolically labeled in order to achieve the steady-state equilibrium of lipid labeling. Cell lipids were extracted and subjected to a two-phase partitioning and gangliosides and non-ganglioside sphingolipids were separated and analyzed by HPTLC. As shown in Figure 2A, in NEU3-HA-GFP expressing cells a 63% and a 75% reduction in GM3 and GD1a ganglioside content, respectively, was observed in comparison to non expressing cells. Interestingly, an increase in GM1 content was also detected in NEU3-HA-GFP expressing cells while GM2 content did not seem to change in the experimental conditions tested. Moreover, a statistically significant increase of lactosyl-ceramide (Lac-Cer), about 43%, was found after NEU3-HA-GFP expression (Figure 2B). No significant variation of the other sphingolipids, i.e. ceramide (Cer), glucosyl-ceramide (Glc-Cer), sphingomyelin (SM) and Gb3, was detected. Changes in the sphingolipid content could be specifically ascribed to the expression of NEU3-HA-GFP since no significant differences in the transcript content of endogenous sialidases, namely NEU1 and NEU3, could be observed in transfected cells grown in presence or absence of dox and compared to non transfected cells (Figure S1).

### NEU3-HA-GFP is associated to both DRM and non-DRM

**Figure 3:**
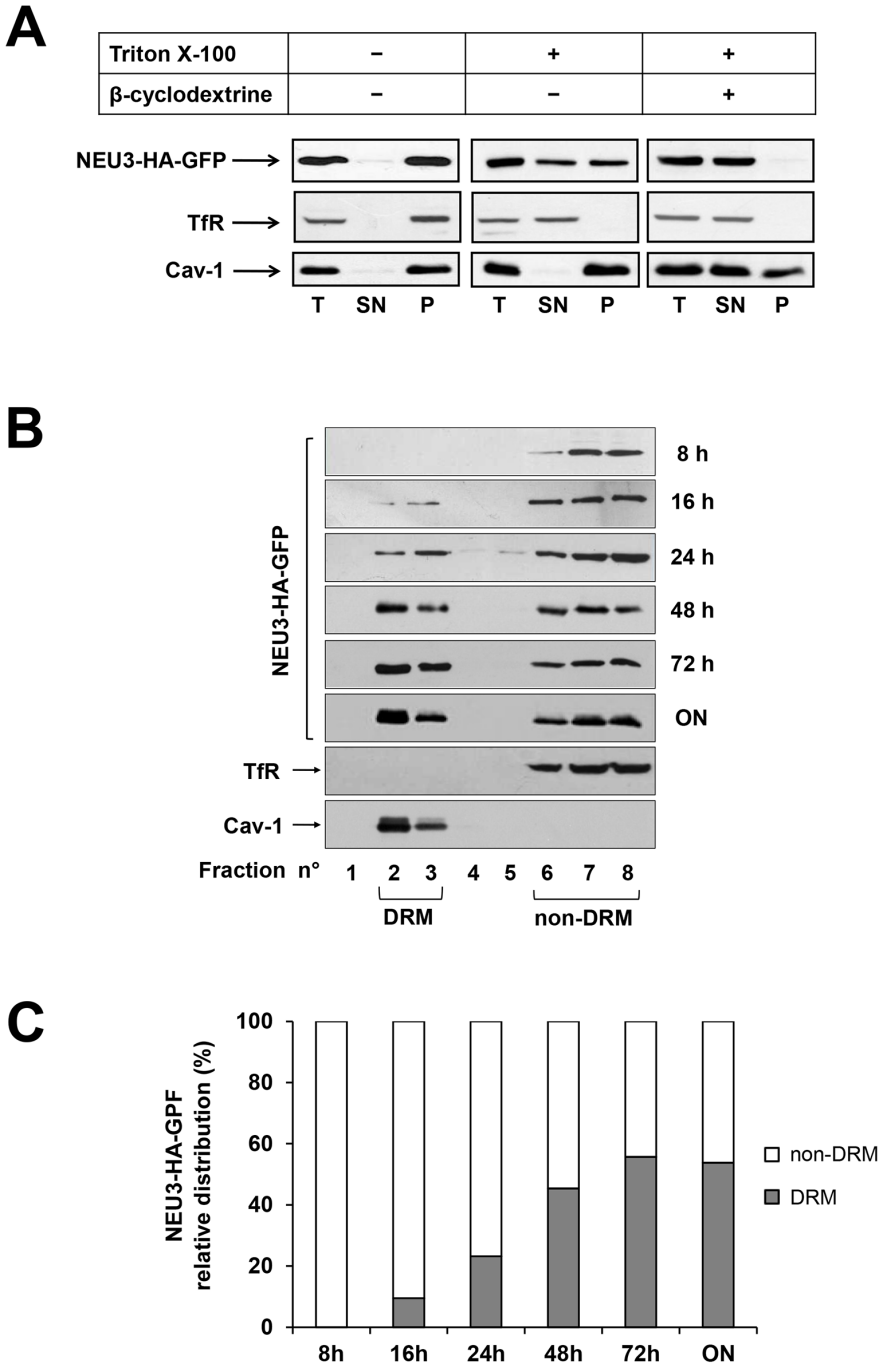
Association of NEU3-HA-GFP to DRM and non-DRM. (A) ON HeLa tTA2 NEU3-HA-GFP grown for 1 h in absence or presence of beta-cyclodextrine were extracted in the appropriate buffer, containing or not 1% Triton X-100 for 30 min at 4°C and ultracentrifuged for 1 h at 100,000×g, Equal amounts of total (T), supernatant (SN) and pelleted (P) material was analyzed by western blot using anti-HA, anti-Transferrin Receptor (TfR) and anti-caveolin-1 (Cav-1) primary antibodies. (B) OFF HeLa tTA2 NEU3-HA-GFP were grown in absence of dox for the indicated time periods and extracted in the appropriate buffer containing 1% Triton X-100 for 30 min at 4°C. DRM and non-DRM were separated by Opti-Prep density gradient centrifugation. Equal amounts of each gradient fraction were analyzed by western blot as indicated in (A). (C) Relative distribution of NEU3-HA-GFP between DRM (fractions 2 and 3) and non-DRM (fractions 6 to 8) referred to the optical density of bands in (B).

In order to study the association of NEU3-HA-GFP to different membrane domains, HeLa tTA2 NEU3-HA-GFP cells were grown for 72 h in absence of dox and extracted for 30 min at 4°C in the appropriate buffer, i.e. either containing or not 1% Triton X-100. Cell extracts were then subjected to ultracentrifugation for 1 h at 100,000×g, and NEU3-HA-GFP distribution between supernatant and pelleted material was compared to the DRM marker caveolin-1 (Cav-1) and the non-DRM marker Transferrin receptor (TfR) (Figure 3A). Treatment with cold Triton X-100 resulted in an almost equal distribution of the enzyme between detergent-insoluble and -soluble fractions, indicating that NEU3-HA-GFP is associated to different membrane subsets that show different sensitivity to detergents, possibly representing DRM and non-DRM, respectively. In order to better understand the mechanism of association of NEU3-HA-GFP to the detergent insoluble membranes, cells were grown for 1 h in presence of methyl-beta-cyclodextrin before harvesting, in order to extract cholesterol from cellular membranes, thus impairing DRM organization. Under these experimental conditions, NEU3-HA-GFP was exclusively detected in the soluble fraction indicating that its association to DRM is dependent on the presence of cholesterol (Figure 3A). As expected for a cholesterol-binding protein such as Cav-1 [20], [21], a partial solubilization of the DRM marker was observed after cholesterol depletion, suggesting the possibility that, at least, a fraction of the protein is associated to DRM in a cholesterol-independent manner [22].

These data demonstrate that sialidase NEU3-HA-GFP is associated to different membrane subsets, i.e. one pool of the protein is associated to detergent-sensitive membranes, and a second pool is associated to detergent-insensitive and cholesterol-sensitive membranes.

### Newly synthesized NEU3-HA-GFP is initially associated to non-DRM

In order to better characterize the membrane subcompartments to which NEU3-HA-GFP is associated, OFF HeLa tTA2 NEU3-HA-GFP cells were shifted to ON conditions for different time periods and the distribution of the enzyme was analyzed after Triton X-100 extraction and discontinuous Opti-Prep density gradient separation. The distribution of NEU3-HA-GFP was analyzed in all gradient fractions and is shown in Figure 3B. As expected, Cav-1 was detected only in the light fractions (fractions 2-3), while TfR was detected exclusively in the dense fractions (fractions 6-8), demonstrating that a correct separation between DRM and non-DRM was achieved. Interestingly, after 8 h expression the newly synthesized NEU3-HA-GFP was recovered exclusively in non-DRM fractions and only after 16 h expression we could detect the protein also in the DRM fractions. Prolonging the NEU3-HA-GFP expression period resulted in the full expression at 72 h and in the equal distribution of the protein between DRM and non-DRM. It is worth to note that the 1∶1 distribution of the protein between DRM and non-DRM fractions is reached already at 48 h expression (Figure 3C). Taken together these data clearly demonstrate that during its biosynthesis NEU3-HA-GFP is initially associated to non-DRM and that the equal repartition of the protein between DRM and non-DRM membrane subcompartments is achieved before reaching the maximum level of expression of the enzyme.

### Newly synthesized NEU3-HA-GFP specifically modifies the ganglioside pattern of DRM and non-DRM

**Figure 4:**
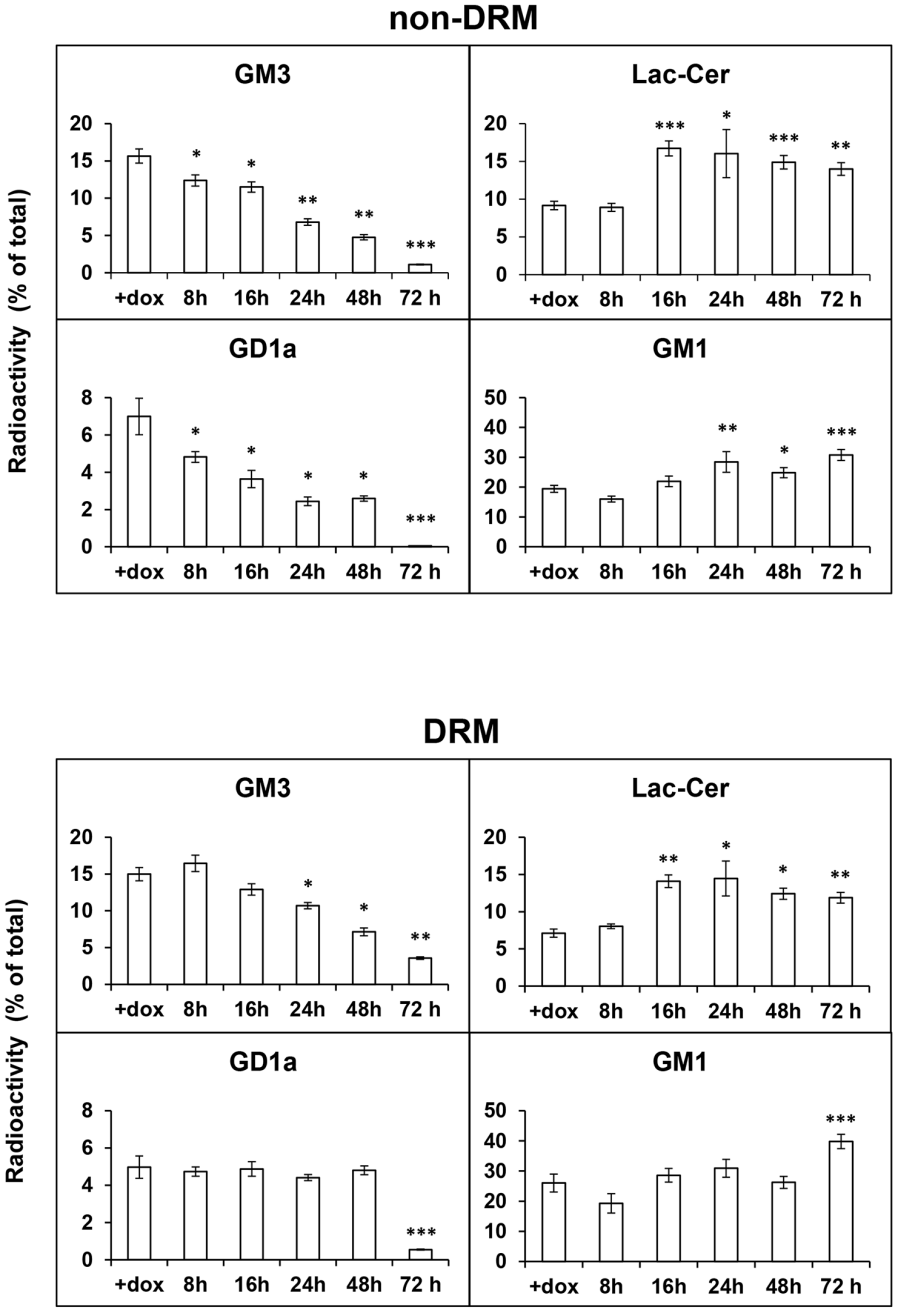
NEU3-HA-GFP specifically modifies the ganglioside pattern of non-DRM and DRM. OFF HeLa tTA2 NEU3-HA-GFP cells were metabolically labeled for 2 h with [^3^H]Sphingosine and, after a 24 h chase, dox was removed for the indicated time periods. Cells were then extracted in the appropriate buffer containing 1% Triton X-100 for 30 min at 4°C. non-DRM (upper panel) and DRM (lower panel) were separated by Opti-Prep density gradient centrifugation. Equal aliquots of fractions 2 and 3 (DRM) and of fractions 6, 7 and 8 (non-DRM) were pooled and gangliosides and non-ganglioside sphingolipids were extracted, separated and quantified. Values are given as percentage of total and represent the means ± S.D. of 3 independent experiments. *p<0.05; **p<0.01; ***p<0.001.

To investigate whether newly-synthesized NEU3-HA-GFP is specifically active toward gangliosides of DRM and non-DRM areas, OFF NEU3-HA-GFP transfected cells were metabolically labeled for 2 h with [^3^H]Sphingosine and, after a 24 h chase, dox was removed and cells were further cultured up to 72 h to allow NEU3-HA-GFP expression. Cells were collected after different time periods, extracted in presence of Triton X-100 and subjected to Opti-Prep density gradient fractionation. Equal aliquots of fractions 2 and 3, representing DRM, and of fractions 6, 7 and 8, representing non-DRM, were pooled and ganglioside and neutral sphingolipids were extracted and separated as described in Methods. We found a significant reduction in the radioactivity associated to GM3 and GD1a in non-DRM fractions already after 8 h expression (Figure 4). This reduction progressively went on with NEU3-HA-GFP expression, reaching extremely low levels of radioactivity associated to GM3 and GD1a after 72 h expression. Correspondingly, a significant increase in Lac-Cer content in non-DRM fractions was observed starting from 16 h, while for GM1 significance was measured after 24 h expression (Figure 4).

In the DRM membrane subcompartment, the radioactivity associated to GM3 slowly started to decrease after 16 h of NEU3-HA-GFP expression (Figure 4), when the protein becomes detectable in these membrane areas. The reduction of GM3 became significant starting from 24 h expression and progressively went on with time. Again, after 72 h expression, the radioactivity associated to GM3 reached very low levels. Lac-Cer content of DRM increased starting from 16 h of sialidase expression, with minor changes during further expression of NEU3-HA-GFP. Radioactivity associated to GD1a and GM1 in DRM did not seem to change significantly up to 48 h expression of NEU3-HA-GFP. Nevertheless, an almost complete loss of radioactivity associated to GD1a and a significant increase in GM1 was observed after 72 h expression of NEU3-HA-GFP. No significant variations in the content of other gangliosides and neutral sphingolipids could be observed, both in non-DRM and in DRM fractions (not shown).

In order to investigate whether the presence of the bulky tag represented by GFP would influence the biological activity of NEU3, we analyzed also the activity of the enzyme using a chimera protein devoid of GFP. For this purpose, HeLa tTA2 cells expressing NEU3-HA under the control of the tetracycline promoter were established. This cell model shared the same characteristics described for the HeLa tTA2 NEU3-HA-GFP model, i.e. expression of the chimera protein is strictly dependent on the absence of dox in the growth medium with a 1.5 higher specific activity and, biosynthesis and degradation followed the same kinetics described for the chimera NEU3-HA-GFP.

When HeLa tTA2 NEU3-HA cells were subjected to [^3^H]Sphingosine labelling and further incubated for different time periods in presence (OFF) or absence (ON) of dox, the radioactivity associated to GM3 and GD1a, both in non-DRM and in DRM subcompartments, decreased in relation to the time intervals of expression of NEU3-HA (Table S1). Correspondingly, an increase in Lac-Cer and GM1 in both subcompartments was observed. Although the overall process of degradation of GM3 and GD1a exerted by the two chimera proteins resulted superimposable, a faster hydrolysis was observed in the case of NEU3-HA. These observations indicate that presence of the GFP-tag does not impair sialidase NEU3 in the recognition of its natural substrates but only temporally delays the enzyme action toward them.

Taken together our data indicate that i) sialidase NEU3 exerts its biological activity toward gangliosides present in the same membrane subcompartments where the protein resides; ii) irrespectively to the tag linked to the enzyme, sialidase NEU3 exerts its catalytic activity toward GM3 and GD1a, both in non DRM and in DRM.

### Sialidase NEU3-HA-GFP is degraded by the proteasome

**Figure 5:**
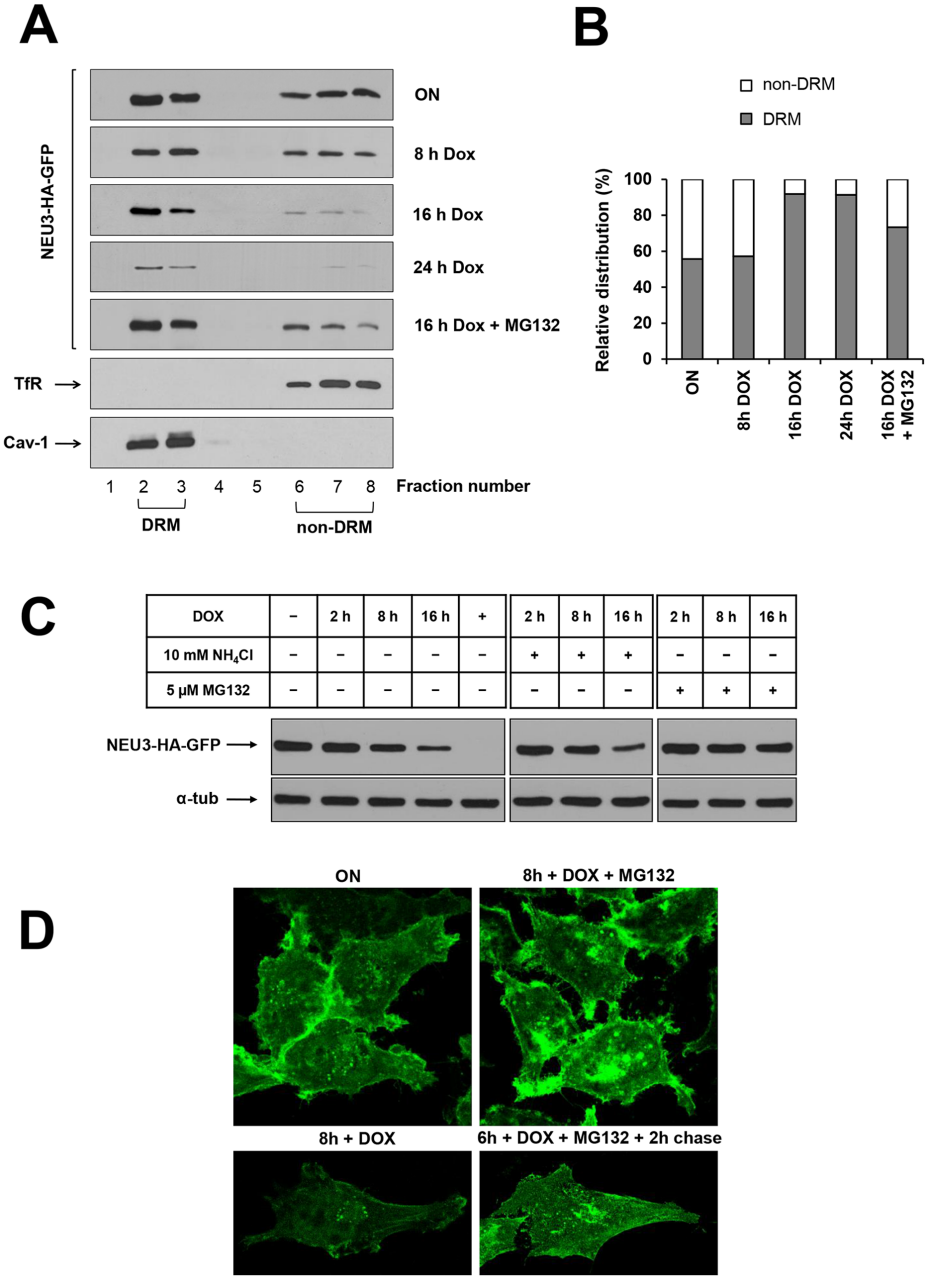
NEU3-HA-GFP is specifically degraded by the proteasomal machinery. (A) ON HeLa tTA2 NEU3-HA-GFP were grown in presence of dox for the indicated time periods, with or without 5 µM MG132, and extracted in the appropriate buffer containing 1% Triton X-100 for 30 min at 4°C. DRM and non-DRM were separated by Opti-Prep density gradient centrifugation. Equal amounts of each gradient fraction were analyzed by western blot using anti-HA, anti-Transferrin Receptor (TfR) and anti-Caveolin-1 (Cav-1) primary antibodies. (B) Relative distribution of NEU3-HA-GFP between DRM and non-DRM referred to the optical density of bands in (A). (C) ON HeLa tTA2 NEU3-HA-GFP were grown in presence of dox for the indicated time periods, with or without NH_4_Cl or MG132. NEU3-HA-GFP expression was analyzed by western blot using anti-HA primary antibody. Alpha-tubulin was detected with specific primary antibody and used as control for total protein loaded on gel. (D) ON HeLa tTA2 NEU3-HA-GFP were plated onto glass coverslips and grown in presence of dox for 8 h, with or without MG132. In order to test reversibility effect of MG132, one sample was incubated for 6 h with MG132, followed by 2 h chase in normal growth medium. After fixation, GFP signal was detected by laser scanning microscope using the same settings (laser power, gain and offset) for all images.

**Figure 6:**
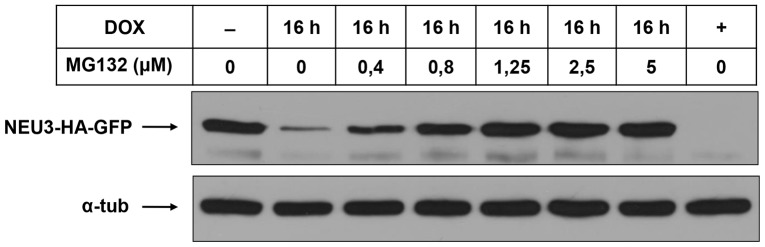
MG132 preserves NEU3-HA-GFP degradation in a dose-dependent manner. ON HeLa tTA2 NEU3-HA-GFP cells were grown in presence of dox for 16 h, with or without MG132 at the indicated doses. NEU3-HA-GFP expression was analyzed by western blot using anti-HA primary antibody. Alpha-tubulin was detected with specific primary antibody and used as control for total protein loaded on gel.

We then analyzed in detail the degradation of sialidase NEU3. For this purpose, ON HeLa tTA2 NEU3-HA-GFP cells were shifted to OFF conditions for different time periods, extracted in presence of Triton X-100 and subjected to Opti-Prep density gradient fractionation. As shown in Figure 5A, incubation for 8 h and 16 h in presence of dox resulted in a fast decrease of NEU3-HA-GFP content. As expected, after 24 h in presence of dox only a residual amount of protein could be detected. In detail, densitometric analysis of the relative distribution of the protein between DRM and non-DRM revealed that, starting from a 1∶1 distribution (ON), after 16 h in presence of dox the residual protein (about 30% compared to ON cells) is almost exclusively present in the DRM fractions in a ratio 9∶1 in favor of light fractions (Figure 5B), indicating that the non-DRM pool of the protein is more sensitive to the degradation process. This distribution is maintained also after 24 h in presence of dox. The relatively short half-life of NEU3-HA-GFP and the availability of a regulated expression system prompted us to investigate which process is responsible for the degradation of the protein. We considered two main protein degradation processes, i.e. the direct lysosomal degradation and the proteasome-mediated degradation. For inhibiting the lysosomal degradative compartment ON HeLa tTA2 NEU3-HA-GFP cells were incubated for up to 16 h in presence of both dox and 10 mM NH_4_Cl. No significant differences in western blot analysis (Figure 5C) as well as in sialidase activity (data not shown) were evidenced in cell extracts deriving from NH_4_Cl treated cells compared to untreated cells, indicating that the lysosomal compartment is not involved in the degradation of the protein. We then analyzed the involvement of the proteasomal machinery and for this purpose ON HeLa tTA2 NEU3-HA-GFP cells were incubated for up to 16 h in presence of both dox and 5 µM MG132, a specific proteasome inhibitor. Cell extracts were tested for their sialidase activity and analyzed for the presence of the protein. Under these experimental conditions, after 16 h in presence of dox and MG132 the enzymatic activity was slightly reduced compared to ON cells (data not shown). As shown in Figure 5C, presence of MG132 in the growth medium significantly prevented NEU3-HA-GFP from degradation, and after 16 h under these experimental conditions the total protein amount was slightly reduced compared to ON cells, indicating that the proteasomal system is responsible for the degradation of the protein. It should be noted that MG132 acts as inhibitor of NEU3 degradation in a dose-dependent manner as shown in Figure 6. Densitometric analysis of the distribution of NEU3-HA-GFP in cells grown for 16 h in presence of dox and MG132 resulted in a 3∶1 repartition of the protein in favor of DRM (Figure 5A and B), further demonstrating that the non-DRM protein pool is more sensitive to the degradation process. Preservation of NEU3-HA-GFP by the proteasome inhibitor MG132 was also confirmed by microscopic analysis taking advantage of the GFP tag. As shown in Figure 5D, after 8 h in presence of dox, a significant decrease in the GFP signal is observed, while cells grown for the same period in presence of MG132 showed an overall signal comparable to ON cells. It should be noted that incubation with MG132 induces a redistribution of NEU3-HA-GFP with a significant accumulation of the protein in intracellular aggregates (Figure 5D). Treatment with MG132 is already known to cause accumulation of different proteins in cytosolic aggregates named aggresomes [23], [24]. Moreover, accumulation of NEU3-HA-GFP in intracellular aggregates was found to be reversible since treatment with MG132 for 6 h followed by 2 h chase in normal medium re-established the normal distribution of the protein (Figure 5D). Overall these results demonstrate that sialidase NEU3-HA-GFP is specifically degraded by the cellular proteasome machinery and that the non-DRM pool of the protein is faster degraded.

### Sialidase NEU3 triggers Akt phosphorylation

**Figure 7:**
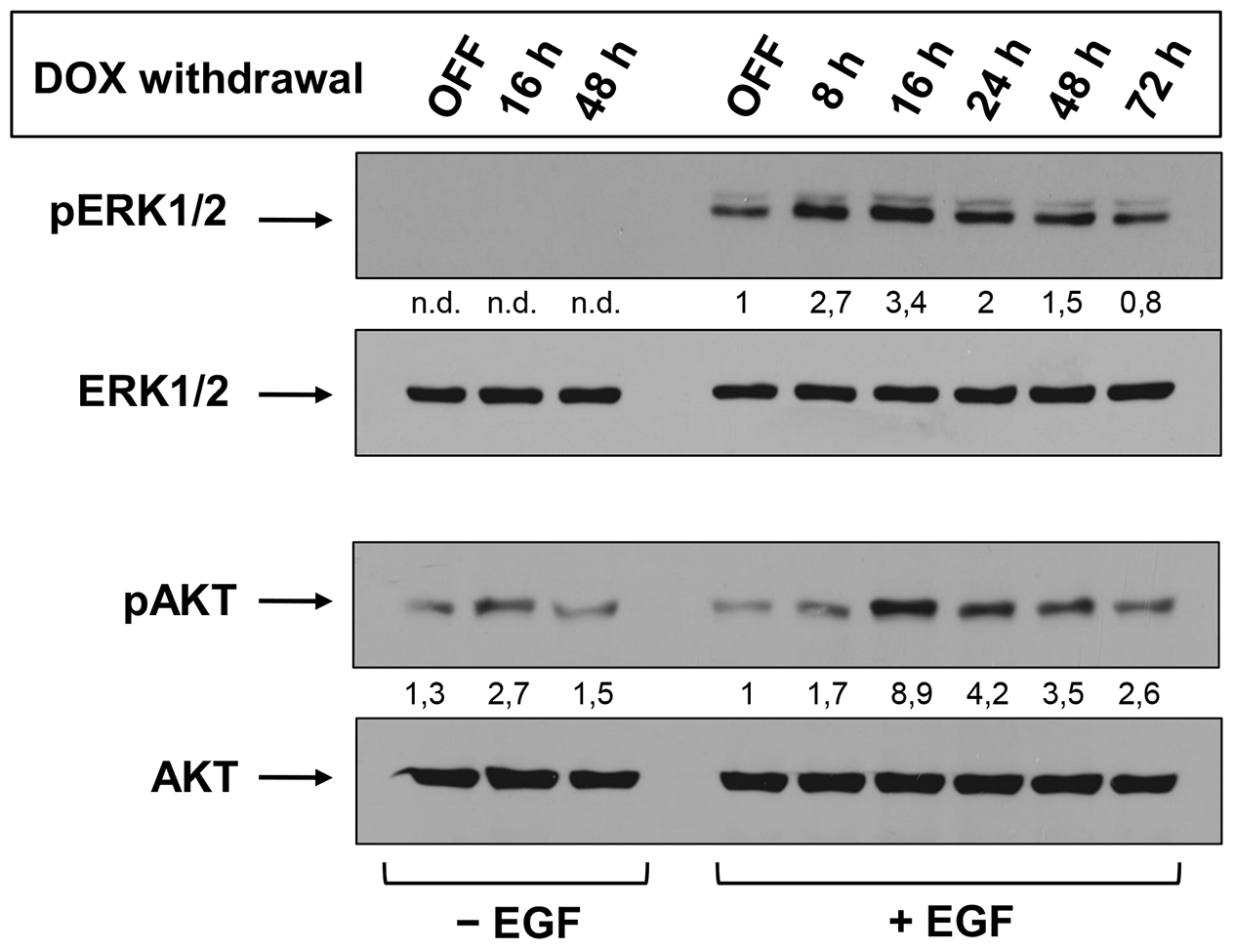
NEU3-HA-GFP triggers phosphorylation of ERK1/2 and Akt. OFF HeLa NEU3-HA-GFP cells were plated and switched ON for the indicated time periods. 24 h before treatment cells were grown in absence of serum. Before harvesting, cells were stimulated or not for 10 min with EGF. Cell extracts were analyzed for pERK/ERK and pAkt/Akt by western blot using specific primary antibodies. pERK and pAkt optical densities were normalized to ERK and Akt, respectively, and values are given.

Overexpression of NEU3 in HeLa cells has been associated to phosphorylation of Ras-downstream molecules [25]. Nevertheless, this information derives from transiently transfected HeLa cells and the analysis of intracellular signaling events was performed only after 48 h transfection, in an end-point approach. Taking advantage of our inducible cell system, the phosphorylation of ERK1/2 and Akt was studied in relation to NEU3 expression levels. OFF HeLa tTA2 NEU3-HA-GFP cells were shifted to ON conditions for different time periods and, before harvesting, stimulated or not for 10 min with EGF. ERK1/2 phosphorylation was found strictly dependent on the presence of the extracellular stimuli (Figure 7, upper panel). Already after 8 h expression of NEU3-HA-GFP, the amount of pERK1/2 is more than doubled compared to OFF cells, with the highest increase detected after 16 h expression. A progressive decrease in pERK1/2 was detected, reaching almost basal levels after 72 h expression. We also analyzed the activation of Akt (Figure 7, lower panel) and found that stimulation of NEU3-HA-GFP expressing cells resulted in a significant increase in pAkt compared to OFF cells. Again, the highest phosphorylation degree was detected after 16 h expression, the time necessary for the appearance of NEU3-HA-GFP in DRM. Interestingly, pAkt was detected also in non-stimulated OFF cells. Moreover, expression of NEU3-HA-GFP in non-stimulated cells was sufficient to trigger phosphorylation of Akt, with a 2.7-fold increase after 16 h expression compared to OFF cells.

These data demonstrate the existence of interplay between NEU3 and pERK1/2 and pAkt, with a particular relevance for Akt pathway, making HeLa tTA2 NEU3-HA-GFP cells a useful model for studying the biological role played by NEU3 in the modulation of signal transduction events.

## Discussion

In late years, the concept of a supramolecular organization of membranes has been well established [2], [26], [27]. This structural organization is represented by membrane areas where specific lipids, such as cholesterol and sphingolipids, are highly concentrated, and the local protein concentration, mainly represented by GPI-anchored proteins, cholesterol-binding proteins, heterotrimeric G-proteins and Tyrosine-kinase receptors, is low [1], [2]. These characteristics brought to the definition of lipid rafts, i.e. transient dynamic assemblies of cholesterol/sphingolipids/proteins [1]. Importantly, the supramolecular organization and composition of cell membranes is a key feature for different biological events such as adhesion, signaling and trafficking [26], [28], [29]. Due to the high concentration in cholesterol and sphingolipids these naturally occurring membrane domains are often referred as liquid-ordered domains [27]. A different concept relating to membrane organization is the Detergent Resistant Membranes (DRM) that identifies membrane structures insoluble after exposure of the membranes to non-ionic detergents at low temperature, also named detergent insoluble membranes [30]. DRM can be isolated from the detergent-solubilized membranes (non-DRM) by density gradient separation. It should be noted that while lipid rafts and liquid-ordered membranes represent physiological entities of the membranes, DRM are formed only after detergent extraction [30]. As already mentioned, cholesterol is known to be enriched in these areas and to play a fundamental and essential role for the existence of DRM [2]. Depletion of cholesterol by incubating intact cells in presence of cyclodextrins results in the disorganization of DRM. Also glycosphingolipids, and gangliosides among them, are highly enriched in membrane domains. In a detergent-free approach, EGFR was found in lipid rafts and the membrane lipid composition may influence the presence of EGFR inside lipid rafts [31]. Moreover, it has been demonstrated that ganglioside GM3 exerts an inhibiting function in EGFR autophosphorylation and, as consequence, activation of intracellular cascade events [5]. In particular, it has been shown that, in compositionally well-defined proteoliposomes, the sialic acid residue linked at the lactosyl-moiety of ganglioside GM3 is the key element for the regulation of EGFR activity [32].

Several lines of evidence demonstrate that overexpression and/or silencing of enzymes involved in the ganglioside degradative pathway can be related to cancer [12], [13], [33]. Sialidase NEU3 is a peripheral membrane protein and plays a crucial role in the regulation of ganglioside composition directly on the cell surface [7] and its involvement in survival of human cancer cells has been demonstrated [25]. In detail, overexpression of NEU3 gives rise to activation of EGFR-downstream signaling, which in turn brings to survival of different human cancer cells. The opposite occurs when NEU3 is down-regulated by siRNA approach. Nevertheless, these results refer to a NEU3 end-point transient expression system and point to the increase in Lac-Cer as the main hallmark for the activation of EGFR signaling. We decided to better characterize the association of NEU3 to cellular membranes, its role in the local modification of the ganglioside pattern and its involvement in cell signaling taking advantage of an inducible-expression system.

Our cell model allowed the expression of the chimera protein NEU3-HA-GFP as a biologically active enzyme, specifically under the tight control of the tetracycline promoter. During its biosynthesis the enzyme is initially found associated to non-DRM (8 h expression) while at full expression time point (72 h) the protein is equally distributed between DRM and non-DRM (Figure 3). NEU3 was found associated to DRM in a cholesterol-dependent manner, suggesting its presence in supramolecular organized membrane structures. The molecular mechanism by which NEU3 becomes partitioned in the two membrane domains remains to be elucidated but it seems unlikely that appearance of the enzyme in DRM results from the complete filling of the non-DRM compartment and consequent transfer of the protein from non-DRM to DRM. Indeed, the one-to-one repartition between the two membrane areas is achieved at 48 h, thus before reaching full expression; prolonging the expression interval to 72 h the sialidase content of both membrane subcompartments is increased. We can speculate that association of newly synthesized NEU3-HA-GFP to DRM may result either from a post-translational modification of the protein that may occur in the non-DRM areas or from the association of NEU3-HA-GFP to other membrane components (protein-protein or protein-lipid association) that may recruit the protein to DRM.

Among sialidases, NEU3 shows high enzymatic specificity toward gangliosides, particularly GM3 and GD1a, both in *in vitro* and in *in vivo* systems [6]–[8], [13], [34]. [^3^H]-Sphingosine labeling of ON and OFF cells demonstrated significant modifications of the ganglioside composition of total cell membranes, particularly in relation to a significant decrease in GM3 and GD1a content (Figure 2). Our cell model allowed also the analysis of changes in ganglioside composition during NEU3-HA-GFP biosynthesis and in relation to the presence of the enzyme in DRM and non-DRM areas. Our results clearly demonstrate that independently from the tag linked to sialidase NEU3, the enzyme can hydrolyze both GM3 and GD1a, regardless to the membrane subcompartment where the protein resides. Hydrolysis of these gangliosides resulted to be faster when sialidase NEU3 was expressed as NEU3-HA chimera instead of NEU3-HA-GFP, particularly toward GD1a present in DRM.

A possible explanation for this delay may reside in a sterical hindrance exerted by the bulky GFP-tag (30 kDa) compared to NEU3-HA (4 kDa). Indeed, presence of the GFP-tag may reduce the possibility of the catalytic portion of the chimera protein in the recognition of the sialic acid moiety of gangliosides, which results in a slower hydrolysis of the gangliosidic substrates. NEU3 has been demonstrated to be a peripheral membrane protein [10], acting “in vivo” toward gangliosides exposed at the plasma membrane of the same cells expressing NEU3 (*cis*-activity) but also belonging to neighboring cells (*trans*-activity) [7]. Presence of the GFP-tag may represent a steric hindrance resulting in the need of a major structural adaptation of the enzyme in the recognition of the glycan moiety to be hydrolyzed.

We could also demonstrate that sialidase NEU3-HA-GFP half-life is about 8 h and its degradation occurs via the proteasome machinery (Figure 5). In detail, in the first 8 h we observed a similar decrease in sialidase signal both in DRM and in non-DRM compartments, giving rise to a decrease of total NEU3-HA-GFP of about 50% compared to ON cells. Incubation of cells for up to 24 h in presence of dox evidenced that the reduction in total sialidase signal can be ascribed to the degradation of the protein from the non-DRM pool. Inhibition of the proteasome for 16 h resulted in a reduction of total sialidase activity of about 25% compared to ON cells that can be ascribed exclusively to the non-DRM pool. Further experiment are needed in order to elucidate whether the non-DRM pool of the protein is more sensitive to degradation events and/or the DRM pool is somehow protected from degradation, maybe by the more rigid and organized membrane environment where the protein resides. To our knowledge this is the first example of a peripheral membrane protein that is degraded via the proteasomal machinery. Nevertheless, the molecular mechanism by which the protein is delivered to the proteasome requires further investigation.

Finally, the phosphorylation state of ERK1/2 and Akt at different time of NEU3-HA-GFP expression and +/− EGF stimulation has been examined. ERK1/2 phosphorylation resulted strictly dependent on EGF stimulation, with a significant increase already at 8 h after NEU3-HA-GFP expression (Figure 7). Activation reached its maximum at 16 h NEU3-HA-GFP expression in presence of EGF, a time point when total sialidase activity is nearly 50% compared to ON cells and almost only 10% of the enzyme is present in DRM. Moreover, by this time an initial decrease in GM3 content in these membrane areas was detected. Similar results were obtained also for Akt phosphorylation in presence of EGF, with an even more pronounced increase in pAkt after 16 h NEU3-HA-GFP expression. Of particular interest is the finding that in EGF non-stimulated cells, expression of NEU3-HA-GFP is sufficient to trigger Akt phosphorylation. Again, even in absence of EGF, pAkt reached a maximum after 16 h of NEU3-HA-GFP expression, a time interval in which the enzyme specifically modifies the sphingolipids pattern of DRM. In this perspective, not only the activity of NEU3-HA-GFP toward gangliosides of different membrane areas results in specific and differential modifications of their sphingolipids pattern, but is associated to modifications of signal transduction cascades. These effects of NEU3 on membrane biology represent a direct link between the enzyme and the complex series of events leading to cancer [12],[35].

In summary, NEU3 represents the first example of a peripheral membrane protein that i) is associated both to DRM and non-DRM, ii) exerts its enzymatic activity toward gangliosides present therein, iii) specifically modifies their ganglioside patterns and iv) is degraded by proteasomal machinery. Moreover, expression of NEU3 not only enhances the phosphorylation state of signaling molecules in response to EGF but its expression is sufficient to trigger phosphorylation of Akt *per se*. Use of proteasome inhibitors/activators and dextrins may represent a possible strategy for regulating the amount of total NEU3 and its presence in DRM and non-DRM. This approach is now under investigation on several tumor cells in our laboratory.

## Supporting Information

Figure S1 **Expression of NEU3-HA-GFP does not influence the expression of endogenous NEU1 and NEU3.** Mock and HeLa tTA2 NEU3-HA-GFP cells were grown for 72 h in presence or absence of dox. Total RNA was extracted and 0.8 µg of RNA were retro-transcribed. Amplification of endogenous NEU1 and NEU3 was performed and the fold change expression of the different genes in NEU3-HA-GFP overexpressing cells compared with Mock cells was normalized to the expression of glyceraldeide 3-phosphate dehydrogenase (GAPDH) mRNA and was calculated by the equation 2^−ΔΔCt^. Values are given as NEU3-HA-GFP relative expression and represent the means ± S.D. of 3 independent experiments.
(TIF)

Table S1 **NEU3-HA modifies the ganglioside pattern of non-DRM and DRM faster than NEU3-HA-GFP.** OFF HeLa tTA2 NEU3-HA-GFP cells were metabolically labeled for 2 h with [^3^H]Sphingosine and, after a 24 h chase, dox was removed for the indicated time periods. Cells were then extracted in the appropriate buffer containing 1% Triton X-100 for 30 min at 4°C. non-DRM and DRM were separated by Opti-Prep density gradient centrifugation. Equal aliquots of fractions 2 and 3 (DRM) and of fractions 6, 7 and 8 (non-DRM) were pooled and gangliosides and non-ganglioside sphingolipids were extracted, separated and quantified. Values are given as percentage of total.
(DOCX)
