# Expression of Concern: Sialidase NEU3 Dynamically Associates to Different Membrane Domains Specifically Modifying Their Ganglioside Pattern and Triggering Akt Phosphorylation

**DOI:** 10.1371/journal.pone.0317802

**Published:** 2025-01-15

**Authors:** The PLOS One Editors

Following publication, concerns were raised about western blot images in this article [1]. Upon editorial follow up, the corresponding author stated there are errors in Figs 1 and 5, and they provided the available underlying data.

Specifically, regarding Fig 1:

- In Fig 1A, the original image was spliced between lanes 2 and 3 of the NEU3-HA-GFP panel, but this was not indicated on the figure. The corresponding author stated that the original underlying blot images are not available; however, data from replicate experiments carried out at the time of the original study have been provided (S1–S2 Files).
- The α-tub panel in Fig 1B is incorrect, and is a duplicate of lanes 2–8 of the α-tub panel in Fig 6. The correct α-tub blot from the original experiment for Fig 1B is provided as Supporting Information (S3 File). Underlying blots for Fig 6 are provided in S4 File.
- The western blot panels in Fig 1C are incorrect. Lanes 1–5 of the NEU3-HA-GFP and α-tub panels of Fig 1C are duplicates of the left NEU3-HA-GFP and α-tub panels of Fig 5C. The experimental conditions for lanes 1–4 are the same for these two experiments, while they differ for lane 5. The corresponding author provided the original underlying data for Fig 5C (S5–S6 Files). The original data are unavailable for Fig 1C; however data from replicate experiments carried out at the time of the original study are provided as Supporting Information (S7–S9 Files).

Additionally, regarding Fig 5:

- The ON panel of Fig 5A is incorrect and is a duplicate of the 72 h panel of Fig 3B. Consequently, the corresponding quantitative band density data for the ON sample in Fig 5B is also incorrect. Here the authors provide a revised Fig 5 in which the western blot panel and quantitative data are replaced with correct data from the original experiment. The original image underlying the replacement NEU3-HA-GFP ON panel in the revised Fig 5A is provided in S10 File, and replicate data for the NEU3-HA-GFP ON panel is provided in S11 File. The underlying quantitative data supporting replicate data for the Fig 5A ON panel is provided in S12 File. The original image underlying repeat data supporting Fig 3B is provided in S13 File.

The corresponding author confirmed that the original individual-level densitometry data and original underlying images are unavailable for Figs 1A-C, 3B (72 h panel), 4, 5A, and 7. They stated that the sialidase enzymatic activity and radioactivity quantitative data of Figs 1 and 2, respectively, are available.

In light of the above issues that raise concerns about the reliability of the published image data, the *PLOS One* Editors issue this Expression of Concern.

**Fig 5:**
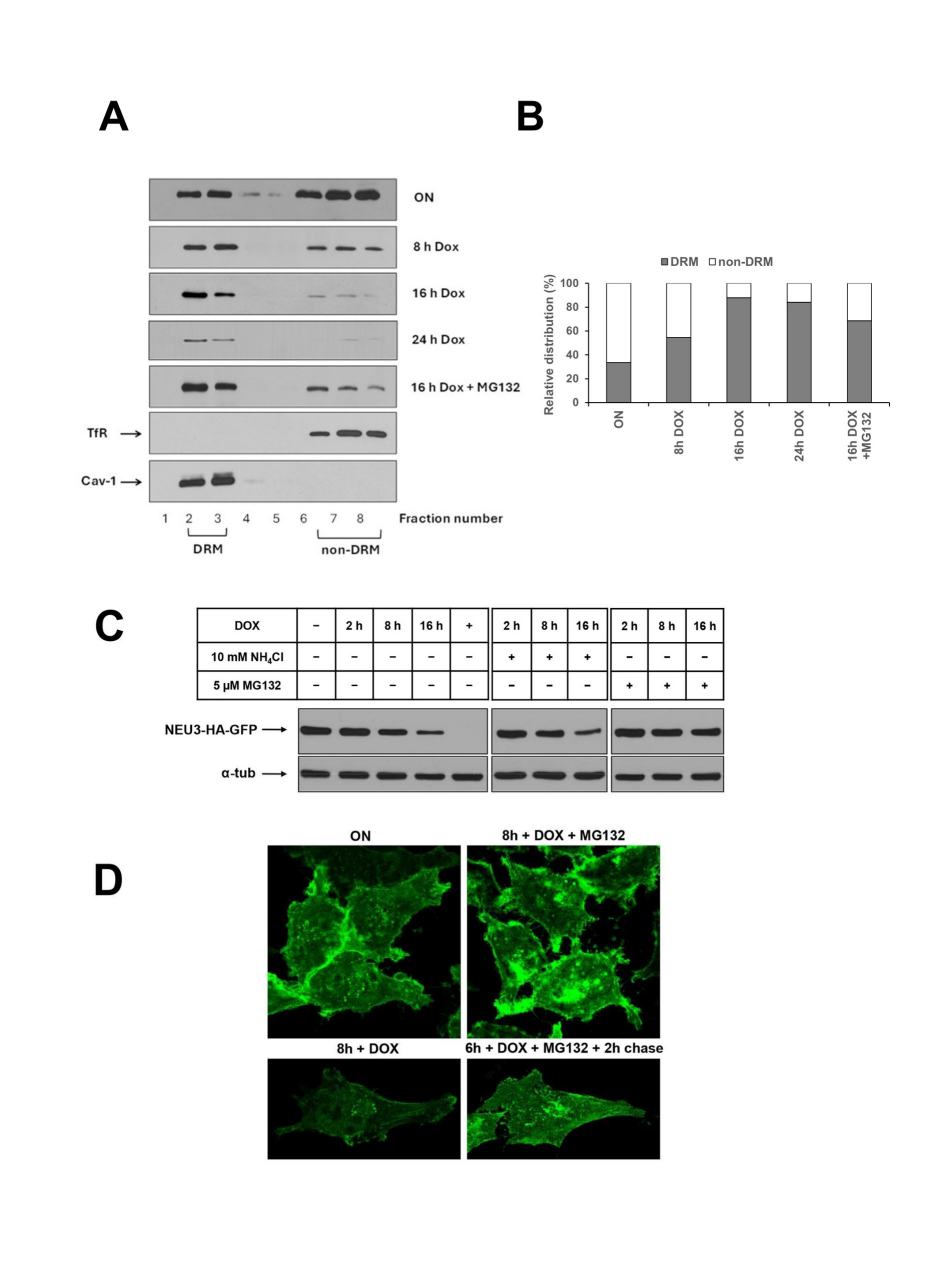
NEU3-HA-GFP is specifically degraded by the proteasomal machinery. (A) ON HeLa tTA2 NEU3-HA-GFP were grown in presence of dox for the indicated time periods, with or without 5 μM MG132, and extracted in the appropriate buffer containing 1% Triton X-100 for 30 min at 4°C. DRM and non-DRM were separated by Opti-Prep density gradient centrifugation. Equal amounts of each gradient fraction were analyzed by western blot using anti-HA, anti-Transferrin Receptor (TfR) and anti-Caveolin-1 (Cav-1) primary antibodies. (B) Relative distribution of NEU3-HA-GFP between DRM and non-DRM referred to the optical density of bands in (A). (C) ON HeLa tTA2 NEU3-HA-GFP were grown in presence of dox for the indicated time periods, with or without NH4Cl or MG132. NEU3-HA-GFP expression was analyzed by western blot using anti-HA primary antibody. Alpha-tubulin was detected with specific primary antibody and used as control for total protein loaded on gel. (D) ON HeLa tTA2 NEU3-HA-GFP were plated onto glass coverslips and grown in presence of dox for 8 h, with or without MG132. In order to test reversibility effect of MG132, one sample was incubated for 6 h with MG132, followed by 2 h chase in normal growth medium. After fixation, GFP signal was detected by laser scanning microscope using the same settings (laser power, gain and offset) for all images.

## Supporting information

S1 File Original image underlying repeat data supporting Fig 1A. (JPG)

S2 File Associated quantitative data for repeat data supporting Fig 1A. (XLSX)

S3 File Original image underlying the correct α-tub panel in Fig 1B. (TIF)

S4 File Original image underlying Fig 6. The left western blot shows the 2 minute exposure and the right shows the 1 minute exposure.
(TIF)

S5 File Original image underlying Fig 5C – 1 minute exposure. (TIF)

S6 File Original image underlying Fig 5C – 5 minute exposure. (TIF)

S7 File Original image for first repeat data supporting Fig 1C. Lane 6 in this repeat data represents the “48h” experiment but in the article it is labelled as the “OFF” experiment, representing the presence of DOX.
(JPG)

S8 File Original image for second repeat data supporting Fig 1C. Lane 6 in this repeat data represents the “48h” experiment but in the article it is labelled as the “OFF” experiment, representing the presence of DOX.
(JPG)

S9 File Associated quantitative data for first and second repeat data supporting Fig 1C. (XLSX)

S10 File Original image underlying the ON panel in the revised Fig 5A. (TIF)

S11 File Original image underlying repeat data supporting the Fig 5A ON panel. (TIF)

S12 File Quantitative data underlying repeat data shown in S11 File. (XLSX)

S13 File Original image underlying repeat data supporting the Fig 3B 72 h panel. (TIF)
