# Supplementary figures and images for: Sialidase NEU3 Dynamically Associates to Different Membrane Domains Specifically Modifying Their Ganglioside Pattern and Triggering Akt Phosphorylation
Source: PLoS One. 2014 Jun 12;9(6):e99405. doi: 10.1371/journal.pone.0099405 (PMC4055604; doi:10.1371/journal.pone.0099405)

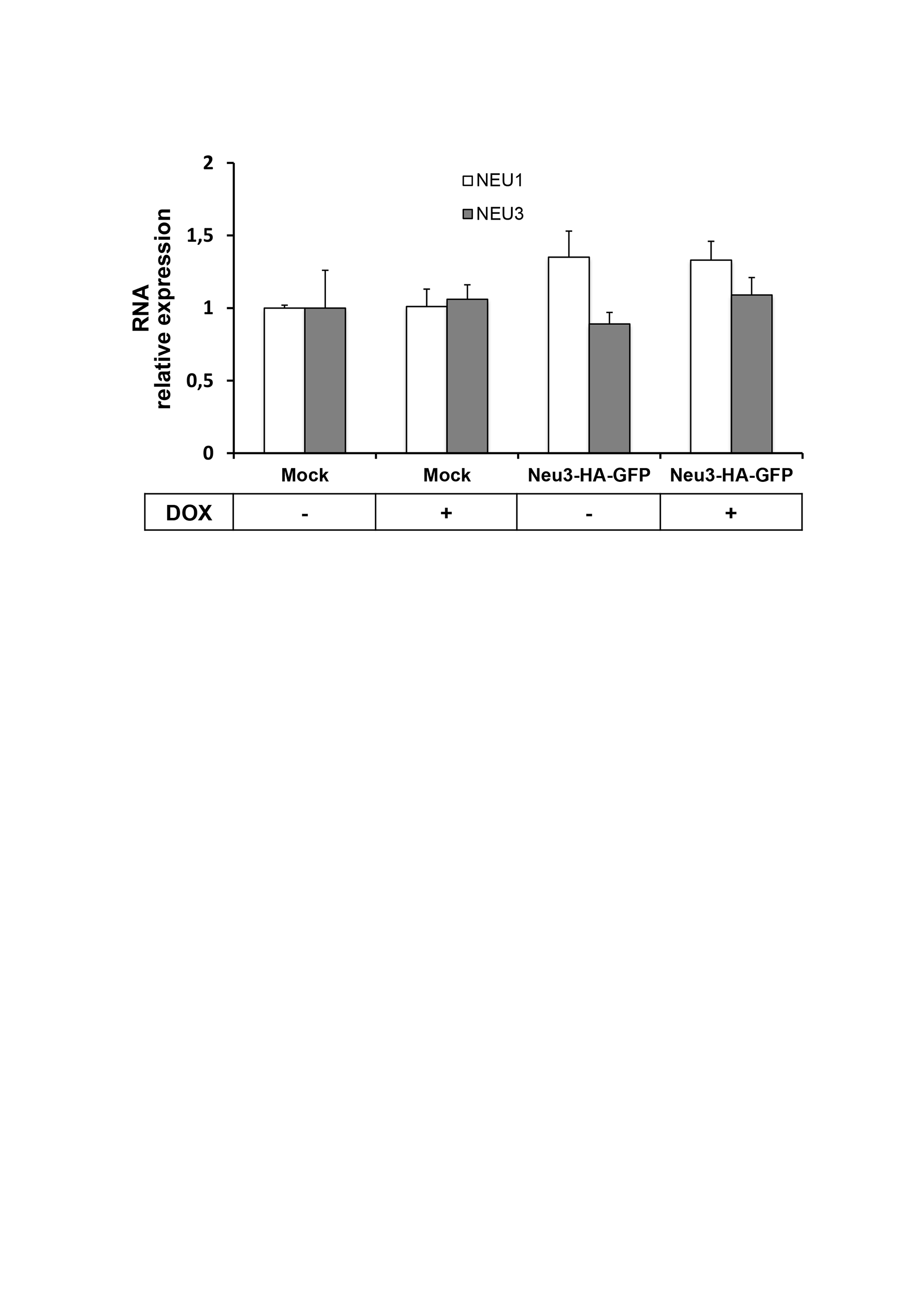

Supplement: Figure S1 — Expression of NEU3-HA-GFP does not influence the expression of endogenous NEU1 and NEU3. Mock and HeLa tTA2 NEU3-HA-GFP cells were grown for 72 h in presence or absence of dox. Total RNA was extracted and 0.8 µg of RNA were retro-transcribed. Amplification of endogenous NEU1 and NEU3 was performed and the fold change expression of the different genes in NEU3-HA-GFP overexpressing cells compared with Mock cells was normalized to the expression of glyceraldeide 3-phosphate dehydrogenase (GAPDH) mRNA and was calculated by the equation 2−ΔΔCt. Values are given as NEU3-HA-GFP relative expression and represent the means ± S.D. of 3 independent experiments. (TIF) [file pone.0099405.s001.tif]
