# Supplementary material for: Sialidase NEU3 Dynamically Associates to Different Membrane Domains Specifically Modifying Their Ganglioside Pattern and Triggering Akt Phosphorylation
Source: PLoS One. 2014 Jun 12;9(6):e99405. doi: 10.1371/journal.pone.0099405 (PMC4055604; doi:10.1371/journal.pone.0099405)
Supplement: Table S1 — NEU3-HA modifies the ganglioside pattern of non-DRM and DRM faster than NEU3-HA-GFP. OFF HeLa tTA2 NEU3-HA-GFP cells were metabolically labeled for 2 h with [3H]Sphingosine and, after a 24 h chase, dox was removed for the indicated time periods. Cells were then extracted in the appropriate buffer containing 1% Triton X-100 for 30 min at 4°C. non-DRM and DRM were separated by Opti-Prep density gradient centrifugation. Equal aliquots of fractions 2 and 3 (DRM) and of fractions 6, 7 and 8 (non-DRM) were pooled and gangliosides and non-ganglioside sphingolipids were extracted, separated and quantified. Values are given as percentage of total. (DOCX) [file pone.0099405.s002.docx]

**Table S1. NEU3-HA modifies the ganglioside pattern of non-DRM and DRM faster than NEU3-HA-GFP.** OFF HeLa tTA2 NEU3-HA-GFP cells were metabolically labeled for 2 h with [^3^H]Sphingosine and, after a 24 h chase, dox was removed for the indicated time periods. Cells were then extracted in the appropriate buffer containing 1% Triton X-100 for 30 min at 4°C. non-DRM and DRM were separated by Opti-Prep density gradient centrifugation. Equal aliquots of fractions 2 and 3 (DRM) and of fractions 6, 7 and 8 (non-DRM) were pooled and gangliosides and non-ganglioside sphingolipids were extracted, separated and quantified. Values are given as percentage of total.

n.d.: not detectable.
