# Supplementary figures and images for: Expression of Concern: Sialidase NEU3 Dynamically Associates to Different Membrane Domains Specifically Modifying Their Ganglioside Pattern and Triggering Akt Phosphorylation
Source: PLoS One. 2025 Jan 15;20(1):e0317802. doi: 10.1371/journal.pone.0317802 (PMC11734951; doi:10.1371/journal.pone.0317802)

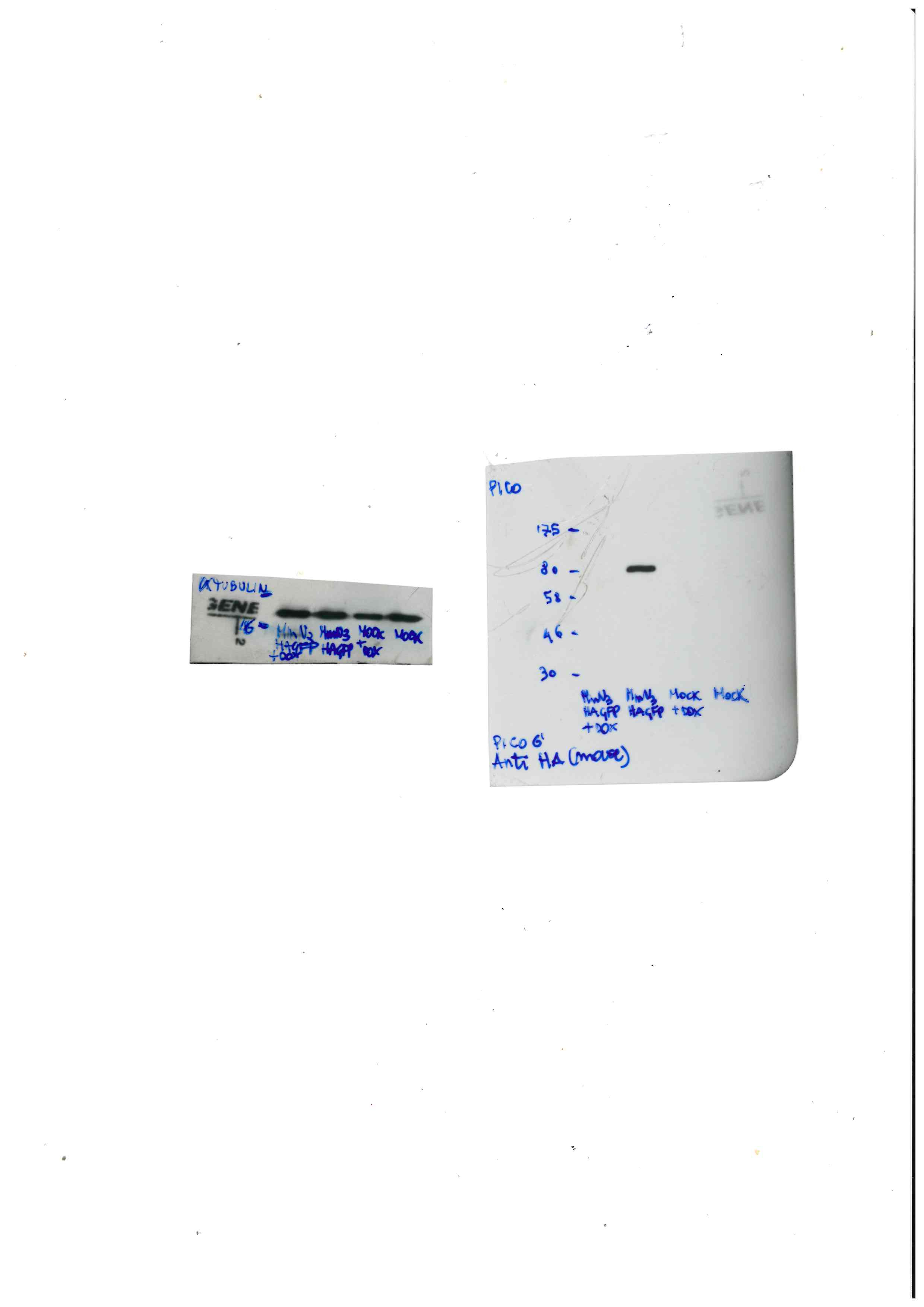

Supplement: S1 File — (JPG) [file pone.0317802.s001.jpg]

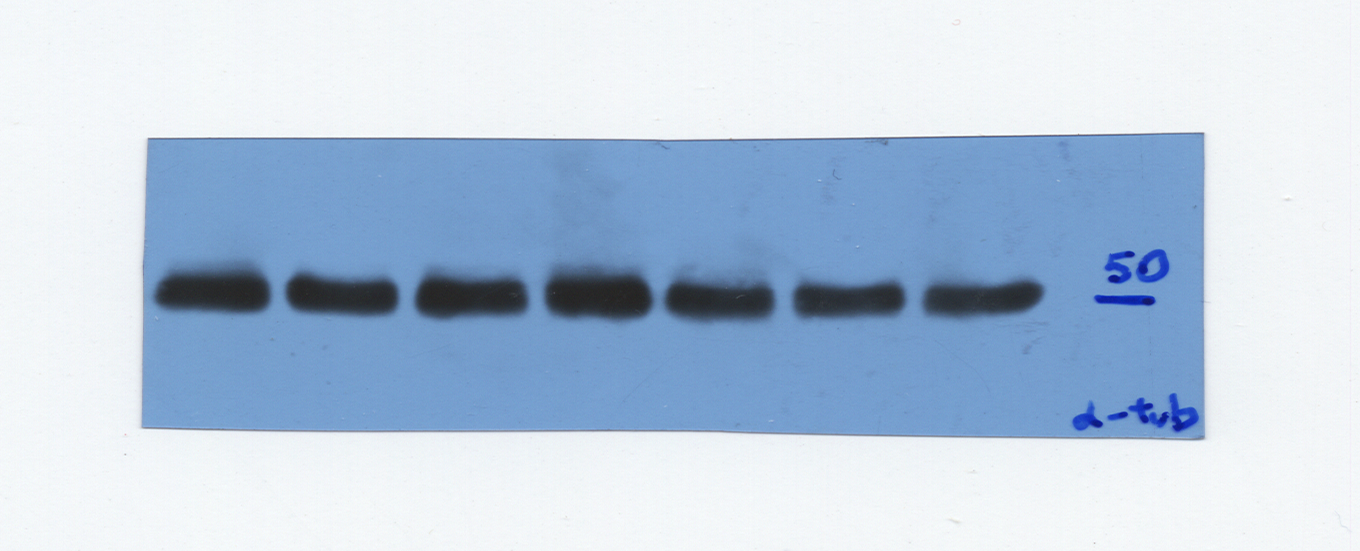

Supplement: S3 File — (TIF) [file pone.0317802.s003.tif]

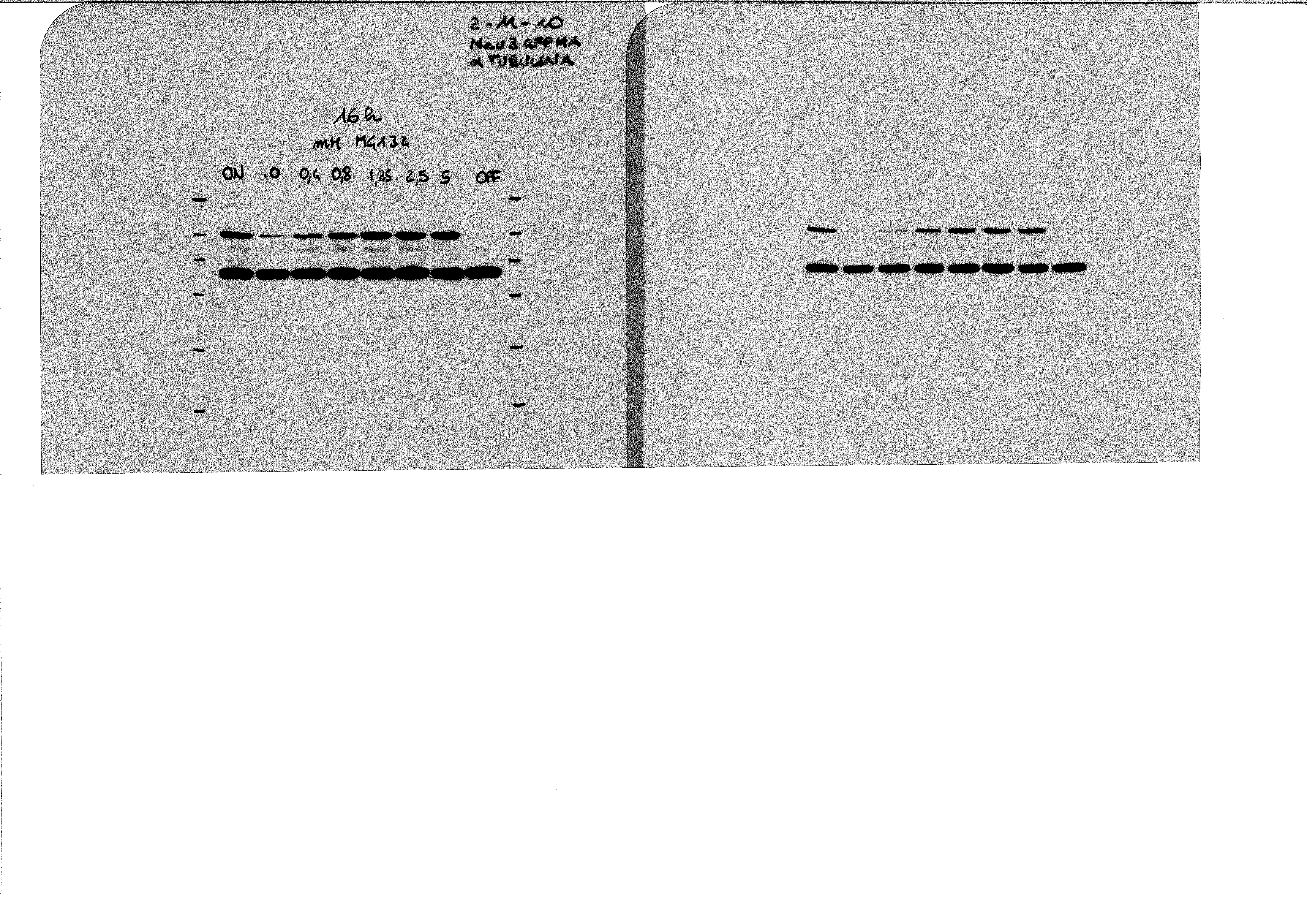

Supplement: S4 File — The left western blot shows the 2 minute exposure and the right shows the 1 minute exposure. (TIF) [file pone.0317802.s004.tif]

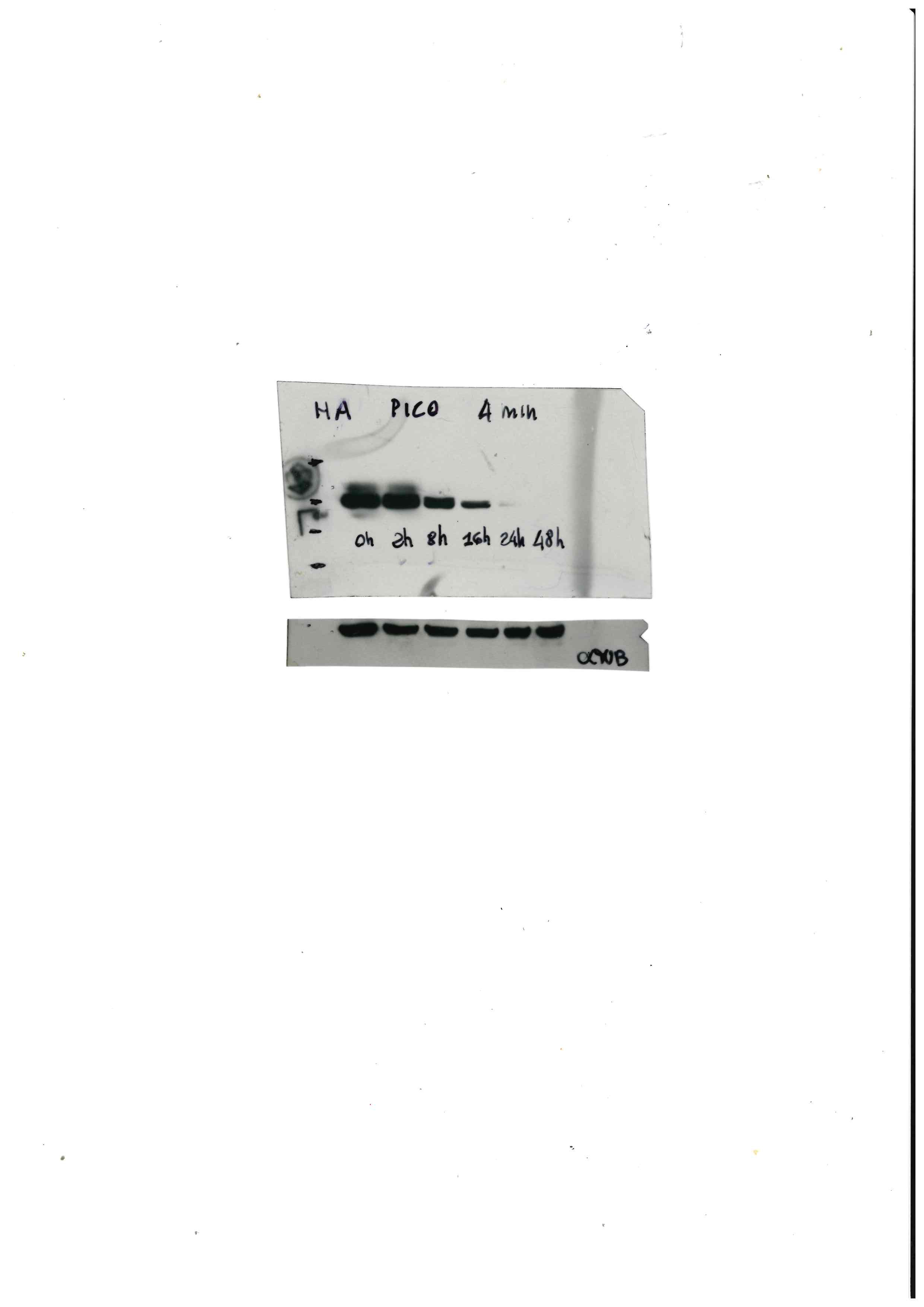

Supplement: S7 File — Lane 6 in this repeat data represents the “48h” experiment but in the article it is labelled as the “OFF” experiment, representing the presence of DOX. (JPG) [file pone.0317802.s007.jpg]

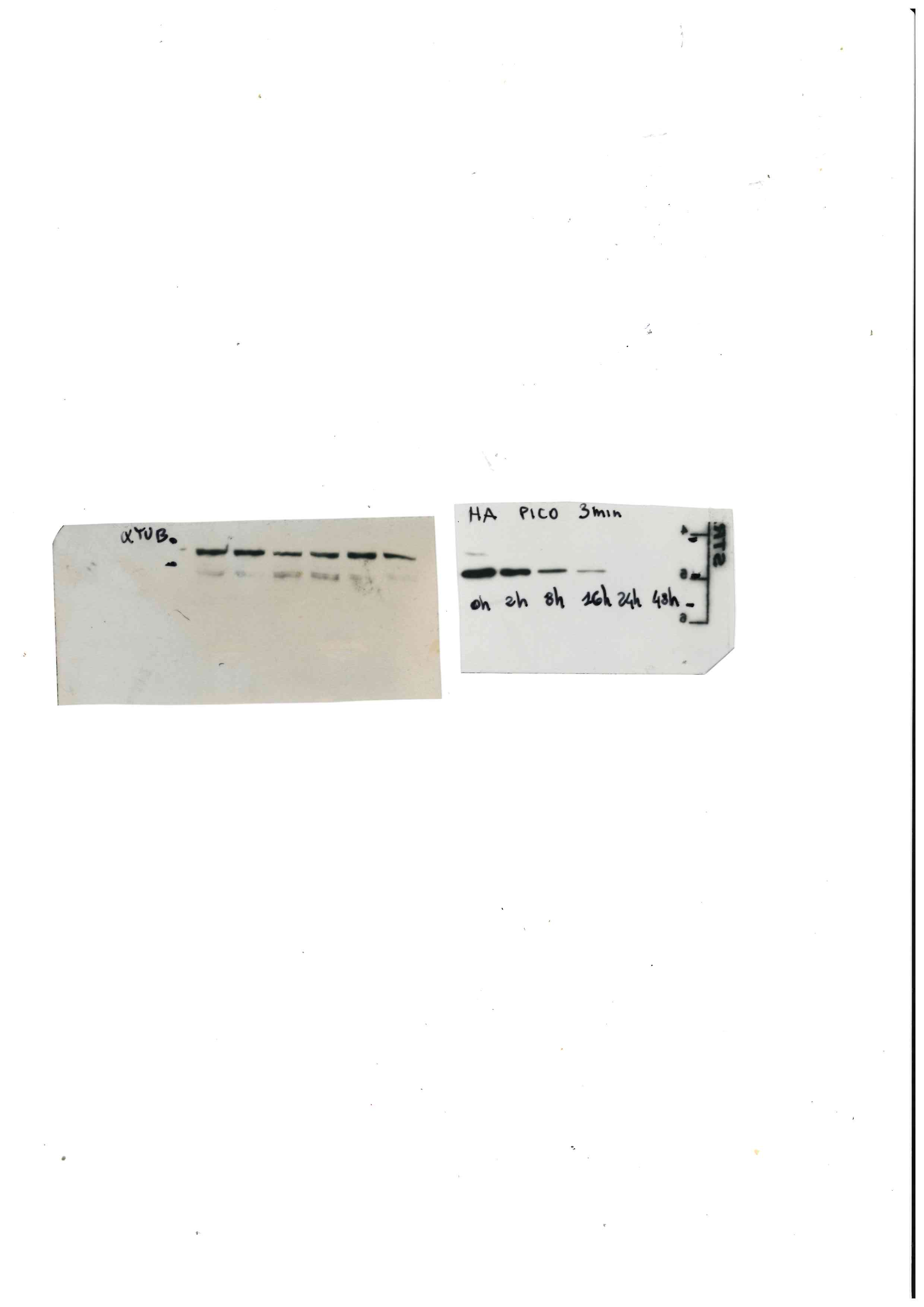

Supplement: S8 File — Lane 6 in this repeat data represents the “48h” experiment but in the article it is labelled as the “OFF” experiment, representing the presence of DOX. (JPG) [file pone.0317802.s008.jpg]

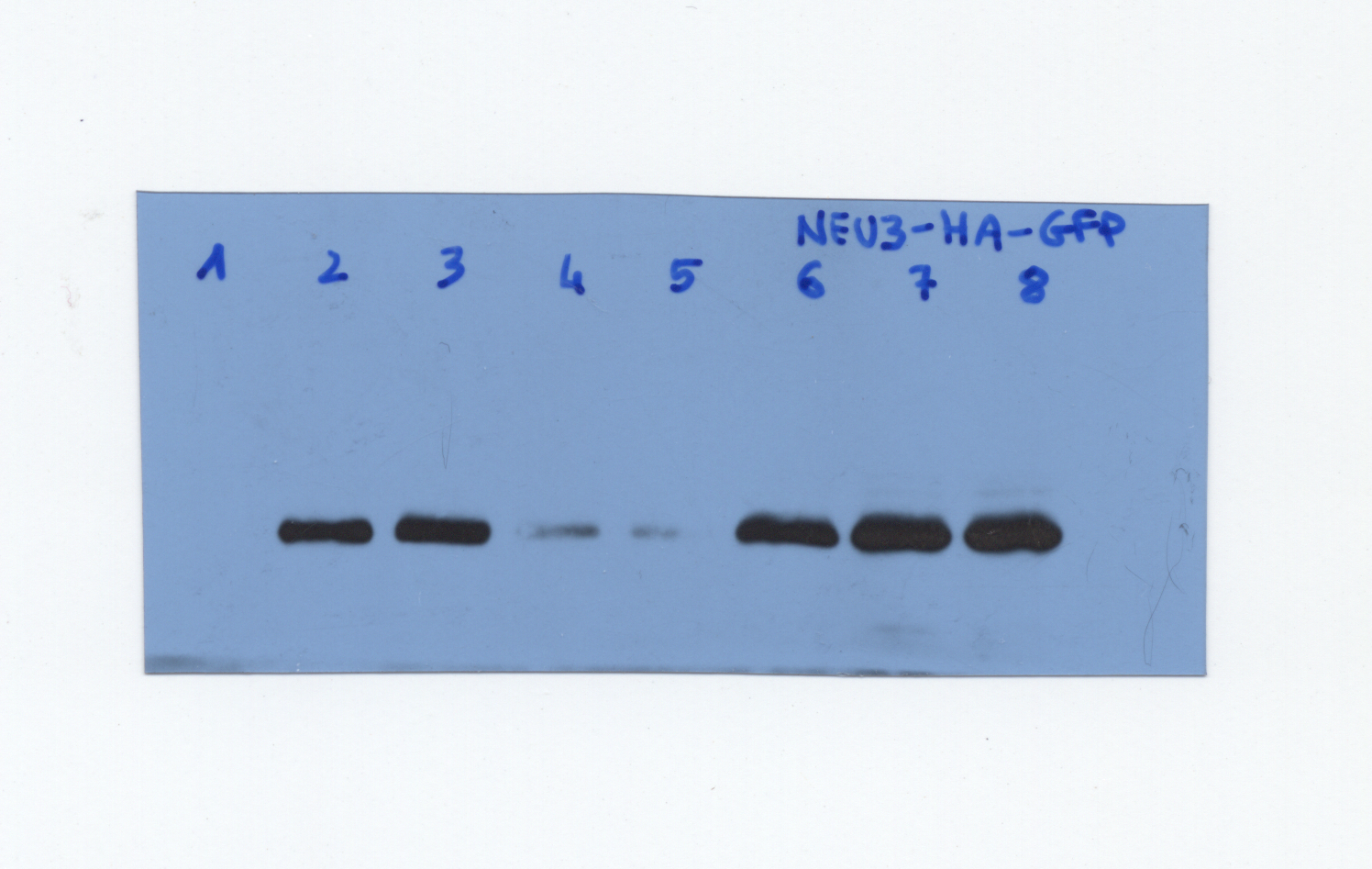

Supplement: S10 File — (TIF) [file pone.0317802.s010.tif]

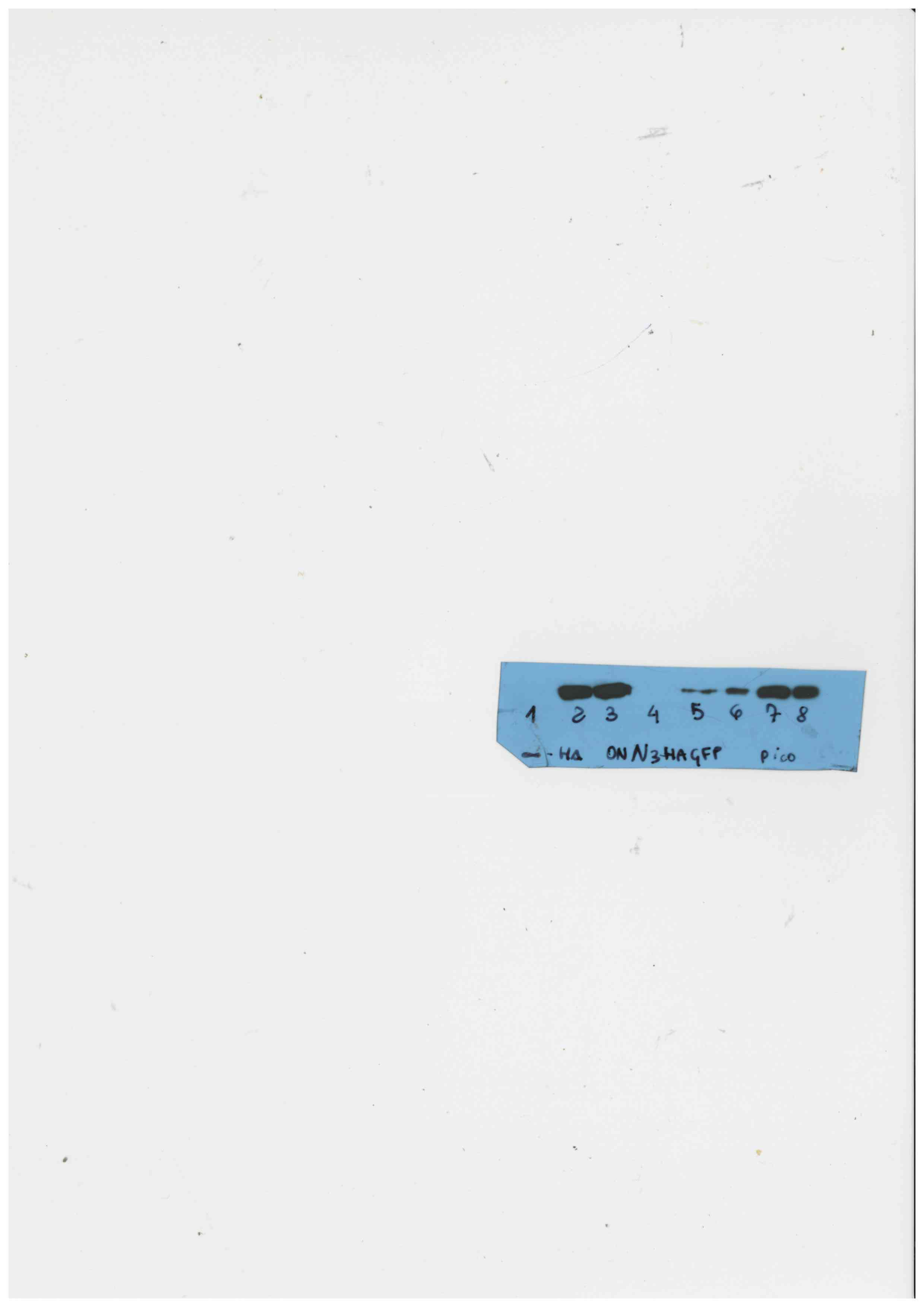

Supplement: S11 File — (TIF) [file pone.0317802.s011.tif]

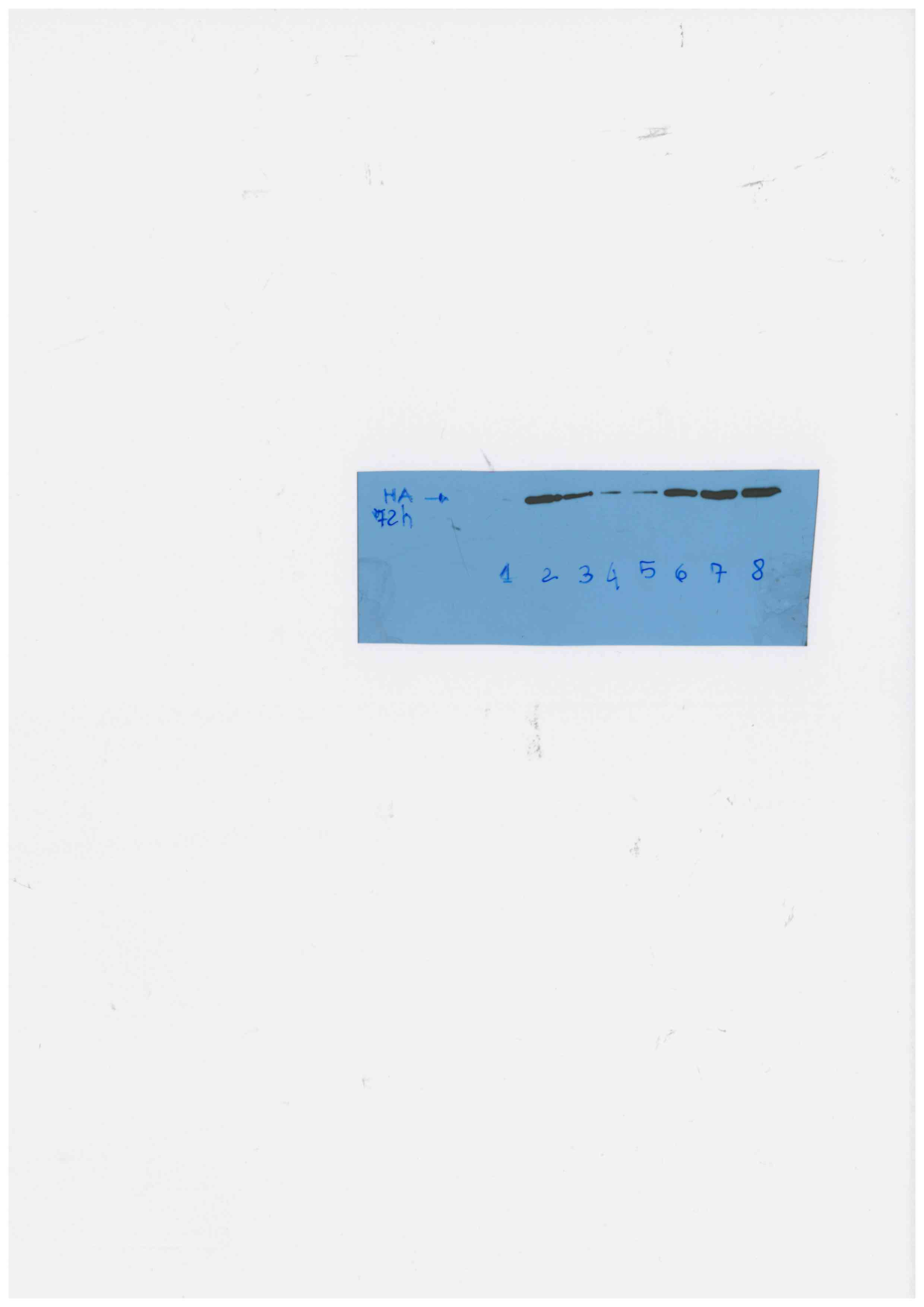

Supplement: S13 File — (TIF) [file pone.0317802.s013.tif]
